# An overview of the benefits of animal-assisted interventions in medical and therapeutic contexts for human health: cognitive mechanisms, sensory perception and welfare considerations

**DOI:** 10.3389/fvets.2026.1757427

**Published:** 2026-03-20

**Authors:** Daniel Mota-Rojas, Eleonora Nannoni, Ana C. Strappini, Ismael Hernández-Avalos, Julio Martínez-Burnes, Adriana Domínguez-Oliva, Patricia Mora-Medina, Ayman H. Abd El-Aziz, Adriana Olmos-Hernández, Agatha Miranda-Cortes, Alejandro Casas-Alvarado, Fabiola Torres-Bernal, Temple Grandin

**Affiliations:** 1 Neurophysiology, Behavior and Animal Welfare Assessment, DPAA, Universidad Autónoma Metropolitana (UAM), Mexico City, Mexico; 2 Department of Veterinary Medical Sciences, DIMEVET, University of Bologna, Bologna, Italy; 3 Animal Health & Welfare, Wageningen Livestock Research, Wageningen University & Research, Wageningen, Netherlands; 4 Facultad de Estudios Superiores Cuautitlán, FESC, Universidad Nacional Autónoma de México (UNAM), Cuautitlán, Mexico; 5 Facultad de Medicina Veterinaria y Zootecnia, Instituto de Ecología Aplicada, Universidad Autónoma de Tamaulipas, Victoria City, Mexico; 6 Department of Animal Husbandry and Animal Wealth Development, Faculty of Veterinary Medicine, Damanhour University, Damanhour, Egypt; 7 Department Bioterio and Experimental Surgery, Instituto Nacional de Rehabilitación Luis Guillermo Ibarra Ibarra (INR-LGII), Mexico City, Mexico; 8 Department of Animal Science, Colorado State University, Fort Collins, CO, United States

**Keywords:** animal-assisted therapy, autism spectrum disorder, dog-assisted therapy, equine-assisted therapy, mental health

## Abstract

Animal-assisted interventions (AAIs) are programs that incorporate animals as a therapeutic factor to improve human well-being. To date, these programs have been associated with significant physical and physiological benefits to human health. Animals have been incorporated as monitoring companions, such as diabetes-alert and seizure-alert dogs. Moreover, animal therapy for people with mental health issues, physical impairments, or autism spectrum disorders has shown social and communicative benefits. Although AAIs are proposed as a complementary treatment approach to humans with mental health issues or to individuals with autism spectrum disorder, further research is needed to address the benefits of AAIs in other medical issues or the instances where animal therapy might not provide a positive effect to patients. This paper provides an overview of the diverse ways in which animals (particularly dogs and horses) can support people, with a special focus on the health benefits they provide. The objective further incorporates the cognitive processes and the ontogenetic development of sensory systems relevant to animal-assisted therapy (AAT).

## 1 Introduction

This paper provides an overview of the wide variety of ways that animals are currently used to help people (particularly dogs and horses). It will be beneficial for both patients and healthcare professionals unfamiliar with AAT and serve as an introduction to the topic. There will be a review of animals that assist with therapy and animals that screen or alert them from different diseases.

Animal-assisted interventions (AAIs) are programs that incorporate animals (e.g., dogs, cats, horses, and even farm animals, birds, and small mammals such as guinea pigs) as a therapeutic aspect to improve human well-being (1–3). The inclusion of animals as assistants in these interventions is due to the benefits associated with positive human-animal interactions. Among these, improving the physical, emotional, and social well-being of humans has been reported (3, 4). It has been reported that interacting with animals increases the concentration of neurohormones associated with a sense of well-being in humans (5–7). AAIs have also shown to strengthen social bonds, regulate mood, enhance motivation, and reduce stress (8, 9), which is why these interventions are proposed and incorporated for patients with emotional or mental disturbances (10–13), or as an emotional support during the recovery from chronic or terminal illnesses (14–16).

AAIs and animal-assisted therapy (AAT) programs have been established in recent decades as a complementary alternative to medical treatments or during the recovery of hospitalized patients (4, 11, 16). Animals also serve as companions to alert certain symptoms in patients with ailments related to hypoglycemia, epilepsy, or cancer (17, 18). Moreover, AAT is frequently adopted in patients with mental health issues (6) as it promotes prosocial interactions, enhancing autonomy, self-esteem, and emotional regulation (16, 19, 20).

In humans with mental health disorders (e.g., schizophrenia, depression, and anxiety) (5, 9), several clinical trials have documented that AAIs contribute to reducing negative symptoms such as apathy, social withdrawal, or anhedonia (21, 22). In depression, positive effects are reflected in mood changes (euthymic mood and the presence of pleasure), especially in older adults (23, 24). In anxiety, immediate benefits include the reduction of symptoms such as tremor, sweating, pale body, and heart palpitations, both in hospitals and in educational settings (8, 25, 26).

AAT has also been adopted in people with autism spectrum disorder (ASD) due to the influence that positive human-animal interactions can have on the communication skills of children and adults (27–29). Studies have reported a decrease in problem behaviors and an improvement in functioning (30). Thus, including animals in healthcare programs can enhance human health and well-being in patients with different conditions (31), which is directly reflected in their quality of life (QoL) (18, 32). Although evidence supports its effectiveness as a therapeutic resource, the incorporation of animals as AAT is still under research (8).

### 1.1 Search methodology

**Figure 1:**
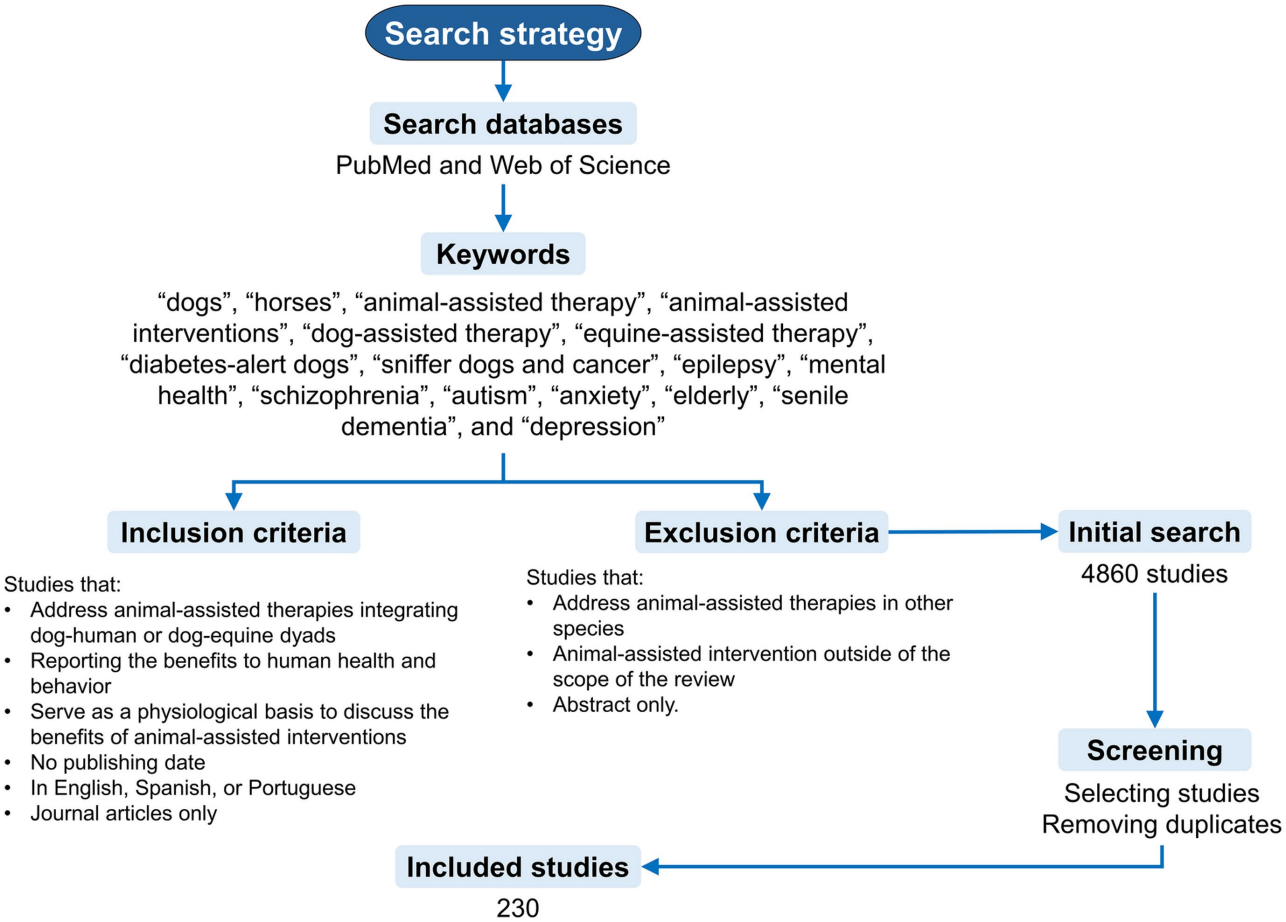
Search strategy.

A literature search was performed using PubMed and Web of Science databases. The search was performed between November and December 2025, using the operators “AND” and “OR.” A combination of keywords containing the following words was used to find studies: “dogs,” “horses,” “animal-assisted therapy,” “animal-assisted interventions,” “dog-assisted therapy,” “equine-assisted therapy,” “diabetes-alert dogs,” cancer detection dogs,” “epilepsy,” “sniffer dogs and “cancer,” “mental health,” “autism,” “schizophrenia,” “depression,” “anxiety,” “elderly,” “senile dementia.” Figure 1 illustrates the search strategy, outlining the inclusion and exclusion criteria for the studies. Studies considered for the review were both original and review papers focusing on studies where dogs and horses were integrated into animal-assisted interventions. The selection of both species was to restrict the number of papers, and because both species have the most studies. No publishing date restriction was established to summarize the published evidence addressing animal-assisted interventions comprehensively. Animal-assisted interventions for individuals with sensory impairments or physical disabilities (e.g., blindness or hearing loss) were considered outside of the scope of the present review to limit the paper to a manageable number of studies. After the screening process, 230 papers were selected and included in the present review.

## 2 What is animal-assisted therapy?

Currently, several worldwide programs incorporate animals in AAIs (3). These interventions include therapies specifically designed for patients with emotional disturbances (10, 11) or psychiatric issues (12, 13), and emotional support during the recovery process from chronic or terminal illnesses (14–16). Service animals, trained to perform specific work or tasks for people with disabilities, are not considered AAI (10).

Howell-Newman and Goldman (33) described the therapeutic use of human-animal bonding to improve the physical and emotional health of patients, considering three categories: animal-assisted activities (AAA), animal-assisted therapy (AAT), and animal-assisted education (AAE).

**Figure 2:**
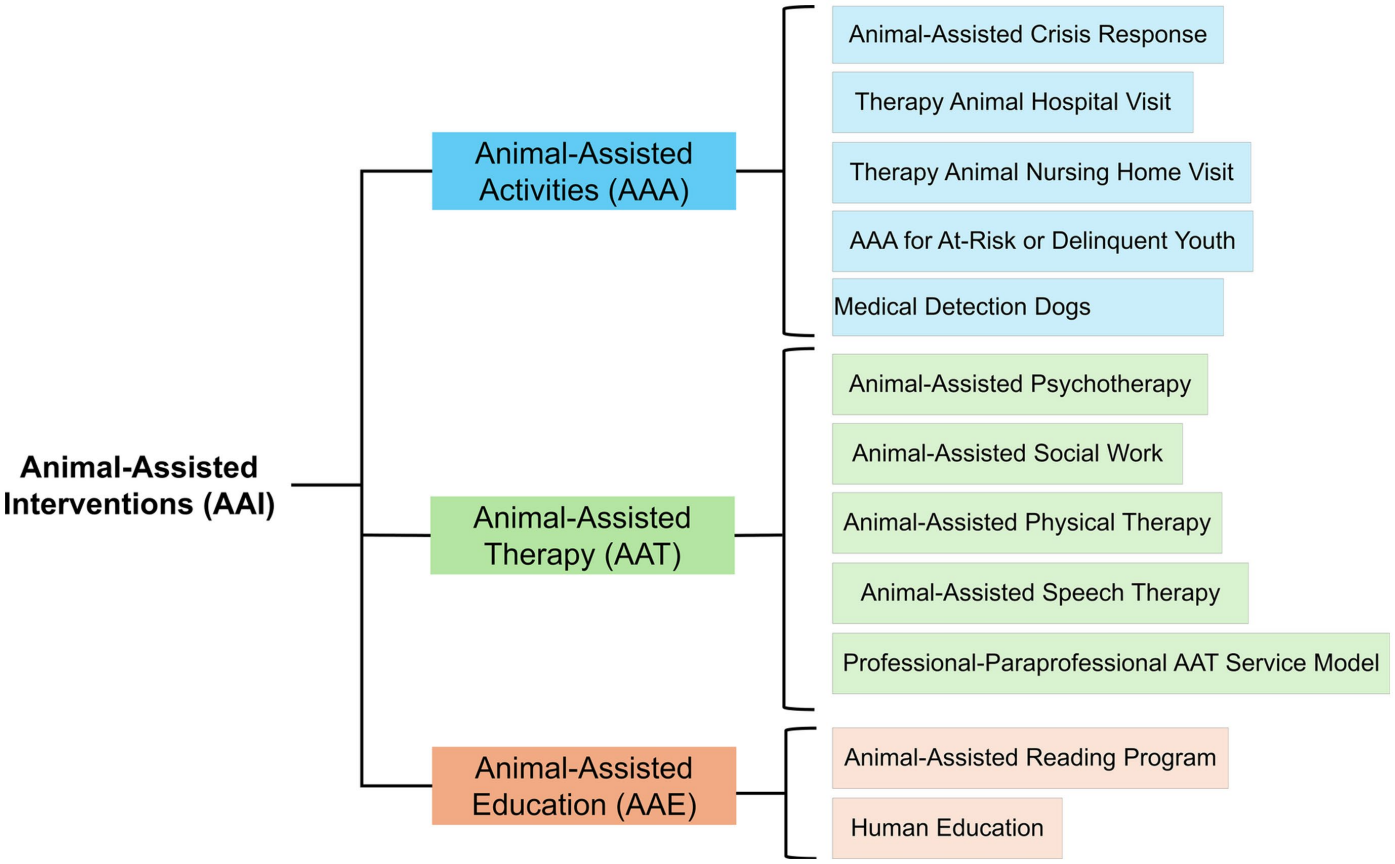
Schematization of the different interventions where humans can interact with animals (15).

AAA are informal interactions with animals that do not have specific therapeutic goals but are designed to promote positive human-animal interaction. In contrast, AAT is a goal-directed therapeutic approach where an animal that meets specific requirements becomes an integral part of a patient’s treatment process. The goal of this therapy is to improve the physical, social, emotional, or cognitive functions of a group or individual (10, 34). AAE comprises the use of animals in educational settings to support learning and personal development (an aspect that is out of the scope of the present review). Figure 2 illustrates he concepts and elements within AAIs, as described by Fine and Mackintosh (15).

Although theoretically any animal species could serve as therapy animals, they are typically registered or certified by assisted intervention organizations after successful training, behavioral verification, and preventive medicine registration. The choice of species will depend on its relational competence derived from its history of coevolution with humans, its ethogram, and its breed (35, 36). Dogs, cats, and horses are the main species used in AAI. Dogs are preferred due to their unique social skills and the close bond developed with humans (37). Moreover, studies have suggested that dogs can interpret social cues from humans (including facial expressions and vocalizations), which translates into support for social communication and interaction (38, 39).

Equines have also shown the ability to establish a connection with humans; therapies involving horses have shown social benefits (40). For example, equine-facilitated psychotherapy is a type of AAT where the sessions are carried out in natural settings or stables. These programs help individuals with emotional and mental health issues, promoting personal exploration of feelings and behaviors, including self-esteem, communication, and socialization (41, 42). It differs from therapeutic horseback riding, which focuses on learning horseback riding (adapted to the rider’s special needs), and hippotherapy (which is therapy using horses and related to physical outcomes) (43).

Farm animal-assisted therapy (FAAT) is another type of AAI that can incorporate donkeys, dairy cows, sheep, goats, rabbits, ducks, and chickens (44–46). It is essentially based on therapeutic interventions where patients interact with domestic farm animals in a natural environment (1, 47). Other aspects that FAAT has been reported to improve are initiative behaviors, attentiveness, and general self-efficacy, as well as strengthening self-esteem in people with clinical depression and anxiety (1, 48), as well as autism spectrum disorders (49). On the other hand, non-conventional animals, such as guinea pigs, have been utilized in occupational therapy and neurorehabilitation to promote fine motor and cognitive skills through meal planning and tasks that include cutting vegetables, hand-feeding, and arranging their living environment (50, 51). Positive effects have also been observed, including the improvement in physical contact and communication, particularly in the quality and quantity of social behavior in autistic children (52).

Therefore, AAT is a therapeutic approach, where the animal is integrated as an active part of the treatment, providing numerous physical, emotional, and cognitive benefits for human. In the following sections, this paper will explore AAI in greater detail, with particular emphasis on AAT.

## 3 The role of animal cognition and sensory perception in AAT

The cognition of animals used in AAIs is a fundamental pillar for the success of the intervention, as it allows them to be active participants. Dogs demonstrate sophisticated social intelligence that is crucial for therapy, including the ability to interpret human signals (53). For example, a therapy dog can discriminate and respond appropriately to gestures, verbal commands, and even subtle changes in the patient’s tone of voice and body language (54). In this regard, Andics et al. (55) identified similar sound-sensitive brain regions in humans and dogs using noninvasive functional magnetic resonance imaging (fMRI). The auditory cortex and subcortical regions, such as the auditory thalamus and the medial geniculate body, exhibited high similarities during the processing of human vocal and nonvocal sounds. Furthermore, dogs exhibited distinct patterns of neuronal activation in response to conspecific and positive human vocalizations, indicating a sensitivity that involves emotional valence during acoustic processing. This scientific evidence is even more relevant when considering the dog’s auditory sensitivity (up to 65 kHz), which is significantly higher than that of humans (up to 19 kHz), encompassing a much wider frequency range and detecting faint or distant sounds (56). This acuity is fundamental in AAIs, as it allows the dog to perceive subtle changes in tone of voice. Within the dog’s social cognitive abilities, it has been shown that canine brain regions such as non-primary auditory regions (the most ventral part of the left caudal Sylvian gyrus and the temporal pole) actively respond to dog-directed speech (exaggerated prosody), suggesting that this quality of human speech is specially tuned in the dog’s auditory cortex to capture their attention and optimize processing (57).

Furthermore, multisensory recognition enables them to interpret nonverbal human signals and adjust their own behavior when interacting with humans in response to the individual’s mood, providing immediate feedback. A region of the dog’s brain, mainly in the bilateral temporal cortex, has been identified as being involved in the perception of faces (both human and canine), demonstrating crucial specialized facial processing during the interpretation of gestures (58). Karl et al. (59) observed significant variations in canine neuronal activity using fMRI. Activation changes were observed in the insula, the bilateral dorsal cingulate cortex, and the postcruciate gyrus in response to a familiar person or human caregiver, which differed from the response to an unfamiliar person (stranger). When subjected to visual preference tests, hippocampal areas such as the left hippocampus showed significantly increased activation in response to happy morph videos, in contrast to angry morph videos, confirming the importance of the human face, especially that of the caregiver, in the dog’s social reward system. A dog’s cognitive ability to decipher human signals or gestures is evident from its early stages of development (60). Dog eye-tracking is likely facilitated by the dog’s evolved ocular morphology. Morphological research has identified a significant anatomical distinction between dogs and wolves, specifically in the periocular musculature (61). Domestic dogs exhibit superior development of the *levator anguli medialis oculi muscle* (AU101), which gives them a greater capacity to retract the upper eyelid, exposing a larger surface area of the scleral tissue and facilitating visual communication with humans (62). High visual discriminative ability has also been observed in AAT horses during social perception. Brubaker et al. (63) observed significant differences during the sociability test in horses interacting with familiar and unfamiliar persons. Horses showed greater preference (spent more time in proximity, approached, and/or made physical contact with the person) for a familiar handler during brushing, which is associated with complex social understanding and emotional sensitivity (64).

Another relevant aspect of animal cognition in AAIs is working memory, which enables the remembering of therapeutic routines and specific individuals, as well as associative learning linking commands with actions (62), all of which are essential during the training of the individual and future work sessions. For example, horses possess specialized socio-cognitive skills and learning abilities (65), as they are gregarious animals capable of learning and imitating the behavior of their conspecifics. This was demonstrated by Evans et al. (66) when applying model-based learning to horses trained using positive reinforcement. The horses showed advanced cognitive abilities and judgment biases when learning to touch a card (with their muzzles) in response to a light cue, which received positive reinforcement. Moreover, particularly when adding negative punishment as the consequence of a negative response, the horses significantly decreased their proportion of incorrect responses (from 16 to 5.5). This implies a developed cognitive understanding, working memory, and the ability to modify behavior to maximize outcomes. This is not exclusive to this species, as dogs have been observed to have a high capacity for episodic memory even when receiving untrained and spontaneous commands without anticipated reward. That is, at least 7 out of 10 dogs responded successfully to the unexpected command even without training (66). This imitative or spontaneous response behavior, without a direct command or prior cue, is strong evidence of a type of memory analogous to episodic memory in dogs (67).

Finally, olfactory cognition constitutes a fundamental neurobiological basis for the dog’s performance in AAT. Dogs have a high olfactory sensitivity (10,000–100,000 times better than that of humans) (68–70), with approximately 200–300 million olfactory receptor cells, in contrast to humans (5 million) (71). Furthermore, a dog’s olfactory cortex covers up to 12.5% of its brain mass (in contrast to the 0.01% in humans (72)), optimizing the processing of olfactory information (73). Thus, dogs can reliably identify specific concentrations of volatile organic compounds (VOCs) emitted by the patient. This sensory capacity is not limited to environmental odors but encompasses the perception of volatile biomarkers that reflect internal physiological states (74–76). This chemical reading of the patient’s state provides the AAT dog with an early multimodal assessment that complements the interpretation of visual and auditory signals.

**Figure 3:**
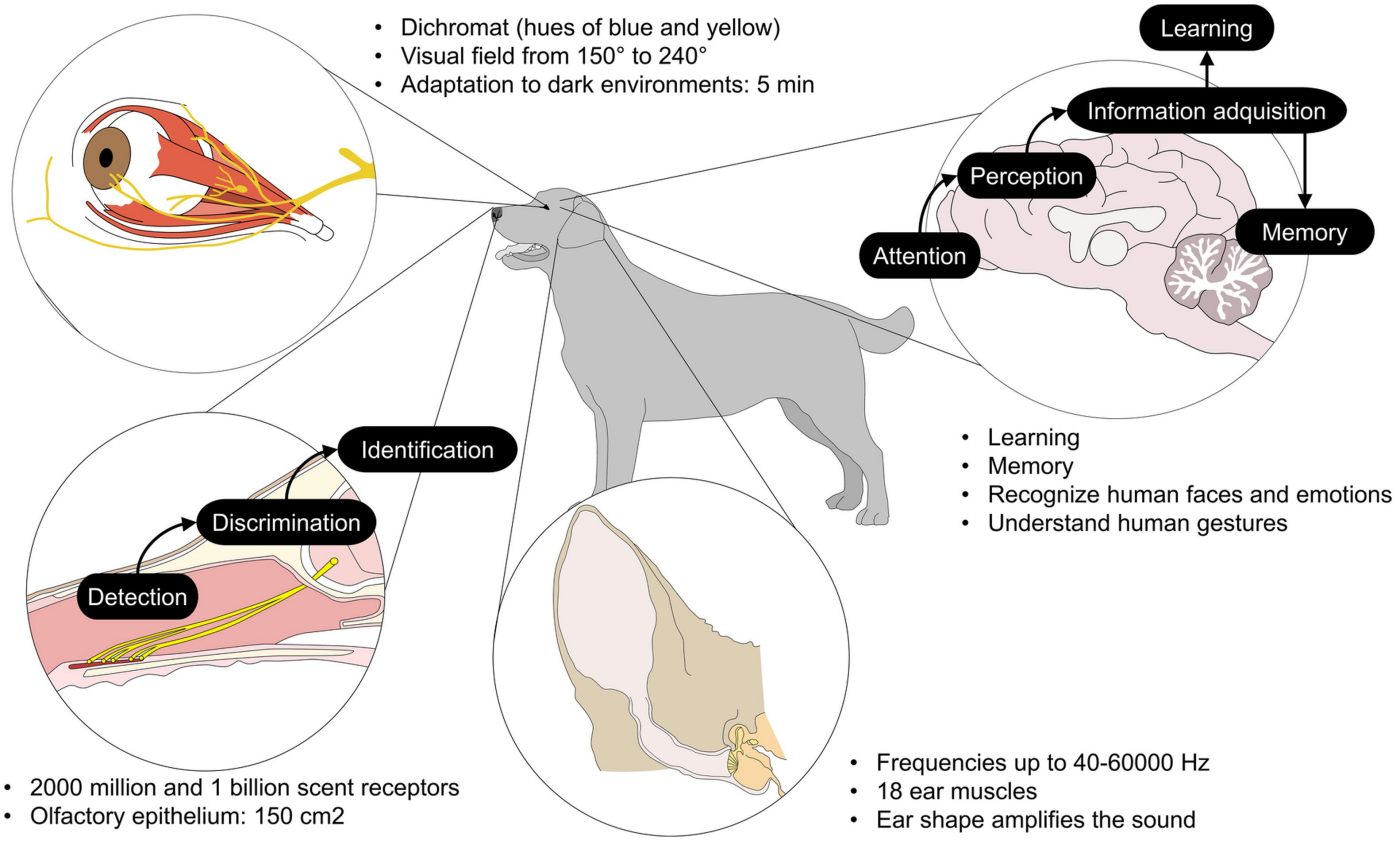
Dog cognition and sensory system. Traits used by animals to participate in AAIs.

AAT leverages dog’s cognitive capacity to create a bidirectional communication bridge that facilitates engagement and the achievement of therapeutic goals. Taken together, these advances confirm that AAT animals are active therapeutic agents, whose ability to perceive, process, and react to human sensory and social information across multiple channels (sight, smell, and hearing) optimizes emotional modulation and the success of interventions. Figure 3 illustrates some of the discussed characteristics of the cognitive and sensory system in dogs (53, 77–79).

## 4 Animal-assisted interventions to screen diseases

Medical Detection Dogs (MDDs) do not meet the strict definition of AAT because they do not offer physical rehabilitation or direct emotional support. However, they are considered within AAI due to their ability to alert biochemical or physiological alterations in humans (such as hypoglycemia or seizures) (80–82), preventing emergencies and mitigating the severity of episodes. Especially in medical assistance roles, dogs act as behavioral and physiological stabilizers, providing a crucial sense of security. By mitigating the threat, the animal enhances the patient’s functional independence, resulting in a comprehensive positive impact on their disease management.

Furthermore, MDDs are trained through a highly specialized process based on the canine olfactory capacity to identify specific VOCs released by the human body in relation to certain health conditions. Currently, the central training mechanism is the association of the odor of a biological sample (sweat, breath, urine, or saliva) from the patient with a reward; that is, operant conditioning and positive reinforcement techniques are used to create an olfactory imprint (83). After odor memorization, training focuses on olfactory discrimination, in which the dog is rewarded only when it correctly identifies and indicates the positive sample. Moreover, a specific alert behavior is finally trained, such as sitting in front of the owner, touching the owner with the nose, among others (84).

### 4.1 Diabetes-alert dogs

Hypoglycemia can be a side effect of insulin therapy in people with type 1 diabetes (84). In mild episodes, daily functioning is impaired, in severe episodes, assistance from another person is required. Neurological and cardiovascular complications might also arise. Over time, patients no longer perceive the symptoms of hypoglycemia, which increases the risk of severe episodes and is associated with higher mortality rates (85). Although glucometers are currently used to monitor human glucose blood levels (84), dogs can serve as a patient-friendly alarm system to noninvasively alert when blood sugar levels fall outside a range (86, 87).

Diabetes Alert Dogs (DADs), a type of MDDs, are animals trained to alert patients with type 1 and type 2 diabetes when their glucose levels are out-of-range (17, 88). These levels generally range from 5 to 15 nm/L (87). Out-of-range (OOR) episodes trigger alert behaviors in dogs through associative learning principles (89). These behaviors include licking the patient, pawing, jumping, staring, vocalizing, and even searching for a blood test kit (87).

The first documented DAD was reported in 2003, with a yellow Labrador Retriever named Armstrong, who was trained to sniff out subtle chemical changes and alert his guardian of an ongoing hypoglycemic episode (17). Later, Wells et al. (90) interviewed 212 dog guardians to document anecdotal evidence that up to 65.1% of non-trained domestic dogs were able to alert at least one of their guardians’ hypoglycemic episodes and showed spontaneous and different attention-seeking behaviors such as vocalizing (61.5%), licking (49.2%), nuzzling (40.6%), jumping on them (30.4%), or staring into the face (41.3%).

Based on empirical evidence (84), several charities began training dogs to alert their diabetic caregivers, as Hardin et al. (84) reported in their evaluation of six dogs that received 6 months of hypoglycemia alert training. During training, sweat samples were collected from patients with hypoglycemia (blood glucose 46–65 mg/dL) and normoglycemia (blood glucose 85–136 mg/dL). These samples were placed in glass vials and then in seven steel cans, only one of which contained the hypoglycemic sweat sample. Dogs were trained to hint at the corrected vial with their muzzle and sit down as an alert sign. The results showed that all dogs had a statistically significantly greater sensitivity (50.0–87.5%) in identifying the hypoglycemic samples than from healthy patients. In a clinical setting, Rooney et al. (89) evaluated the correlation between the behavioral response of 27 DADs and the owners’ glucose levels. The DADs showed median sensitivities of 83 and 67% for hypoglycemic and hyperglycemic episodes, respectively, while the mean sensitivity for OOR episodes was 70%. These results show that DADs can recognize and alert their guardians to an upcoming episode. However, these results may be biased because the data are based on owner reports, which could lead to undetected false negatives. Wilson et al. (85) used a FreeStyle Libre Flash Glucose Monitoring System (FGMS) to monitor glucose levels in patients with DADs. The authors found that DADs were more sensitive during hypoglycemic episodes (55.9%) than during hyperglycemic episodes (36.5%). However, individual variations were observed in dogs’ sensitivity (ranging from 33.3 to 91.7%). Therefore, it is essential to conduct further research to assess the variability observed in DAD responses.

### 4.2 Sniffer dogs and volatile organic compounds of cancerous cells

A global human cancer statistics estimates 20 million cases worldwide in 2022. The most common are lung (12.4%), breast (11.6%), and colorectal cancer (9.6%) (91). Prompt diagnosis of cancer substantially reduces the application of highly invasive interventions and the incidence of metastasis, increasing the probability of survival in human and animal patients (92). Current diagnostic tools for cancer detection include imaging (radiography and ultrasonography), blood and urine analysis, cytology, and histopathology. However, these techniques are expensive and, in some cases, invasive, therefore requiring sedation. Therefore, low-cost, noninvasive diagnostic tools for neoplasia screening, such as MDD, represent sophisticated and essential strategies in oncology (93, 94).

Olfactory detection of the presence of cancerous cells by MDD dogs is due to the high sensitivity and specificity in identifying volatile organic compounds (VOCs) emitted by malignant tumors (75, 95). These aromatic compounds can be found in exhaled breath, urine, saliva, sweat, blood, and feces, among others. They are the result of metabolic changes induced by oxidative stress during neoplasia development and tissue damage. For example, ethane and hydrocarbons are VOCs of lipid peroxidation found in lung cancer (96).

In 1989, a case report described a 2-year-old male Labrador that was non-trained constantly sniffing a 2-cm mass on its owner’s leg. The pet’s fixation prompted the owner to perform a routine medical check-up that resulted in the early detection of a malignant melanoma diagnosed by histology (94). Studies have demonstrated dogs’ ability to differentiate samples from individuals with malignant tumors in both conspecifics and humans. In the case of conspecifics, Malone et al. (93) used MDD (mean of 5.4 years) to differentiate saliva samples from dogs with malignant tumors from those of healthy controls. The results showed that dogs discriminated samples with 90% sensitivity and 98% specificity. In humans, a 2-year-old Beagle was trained to differentiate blood serum samples from patients with newly diagnosed non-small cell lung cancer achieving a sensitivity of 96.7% and specificity of 97.5% (93). In particular, the authors highlighted the importance of the breed, as Beagle show a higher expression of olfactory receptors (225 million) in the olfactory epithelium than other breeds such as Dachshunds (125 million) and Fox Terriers (147 million) (71, 97). These results are similar to those reported by Feil et al. (98) for a 7-year-old Golden Retriever trained to discriminate (using urine and breath samples) between healthy and lung cancer patients. The dog recorded an overall discrimination rate of 97.6%, corresponding to 40 of 41 positive responses in cancer samples, with higher efficiency in urine samples (87.8%) than breath samples (78%). Thus, these results suggest that Golden Retrievers have high olfactory sensitivity (99).

For other types of cancer, it has been shown that two trained female Belgian Malinois dogs, aged 1 and 7 years, could also discriminate cell culture medium or salive samples from osteosarcoma patients with sensitivities and specificities of 95–100% (100). The findings showed higher learning speed and discrimination ability in the young dog compared to the geriatric dog (sensitivities of 97.65 and 90.90%, respectively). Age is associated with brain neuroplasticity (101), which is high during the sensitive period (the first 2 years of life). Therefore, both the breed and age of the dog could influence the sensitivity and specificity of cancer biomarker identification.

A number of studies have documented that trained dogs can successfully olfactory identify VOCs from cancers such as prostate, melanoma, ovarian, colon, and bladder, with generally high sensitivities and specificities, although this varies depending on the study design, as summarized by Pellin et al. (102). However, the literature also reports methodological challenges, including limited reproducibility, variability in training protocols, and small sample sizes (103). The highly accurate canine olfactory capacity makes dogs a promising biomodel to alert the presence of cancerous cells. Nonetheless, protocol standardization, sample expansion, independent replication, and evaluation of diagnostic potential versus conventional methods are required. Moreover, regardless of the precision of their sense of smell, further research is needed to establish whether dogs can differentiate between different types of cancer.

### 4.3 Seizure-alerting and seizure-response dogs

One of the most significant issues regarding seizures (a neurological disease that affects approximately 1% of people worldwide) is the unpredictability of the episodes (80, 104). Currently, devices are under development to monitor and forecast epileptiform activity in the pre-ictal (before) or ictal (during a seizure) phase (105, 106). However, dogs have been shown the ability to alert or report seizures before their onset (81). There are two types of dogs: seizure-alerting dogs (SADs) and seizure-response dogs (SRDs) (32). SADs are specifically trained to successfully alert their owners before a seizure starts (32). These dogs might be trained to activate an alarm, warn the owner/caretaker to anticipate seizures, or wear a backpack with a contact number and medications necessary for the human companion (32, 107). On the other hand, SRDs adopt specific behaviors before, during, or immediately after a seizure, and might provide emotional support at the pre-ictal and post-ictal stage (18, 32, 107). Even without special training, some dogs can spontaneously anticipate their owner’s seizures (108). In this sense, untrained pet dogs have also shown an innate ability to alert their owners before the onset of epileptic activity, and studies have shown that both trained and pet dogs can alert to complex partial and tonic–clonic seizures (32, 109, 110).

Several studies have reported different alert times for both SADs and SRDs. For example, for SADs, a systematic review concluded that the alert time before a seizure ranged from 45 min to a couple of seconds after clinical seizure onset (111), with an accuracy above 70% (107). Other studies report that anticipatory behavior began 60 min to 5 h before the onset of the seizure, with owner-reported accuracy of 70–85% (32, 111, 112). Martinez-Caja et al. (108) reported that 63% of trained dogs anticipated a seizure 30–60 min before its onset, whereas up to 36% of non-trained dogs anticipated seizures 0–10 min before the onset of epileptic activity.

**Figure 4:**
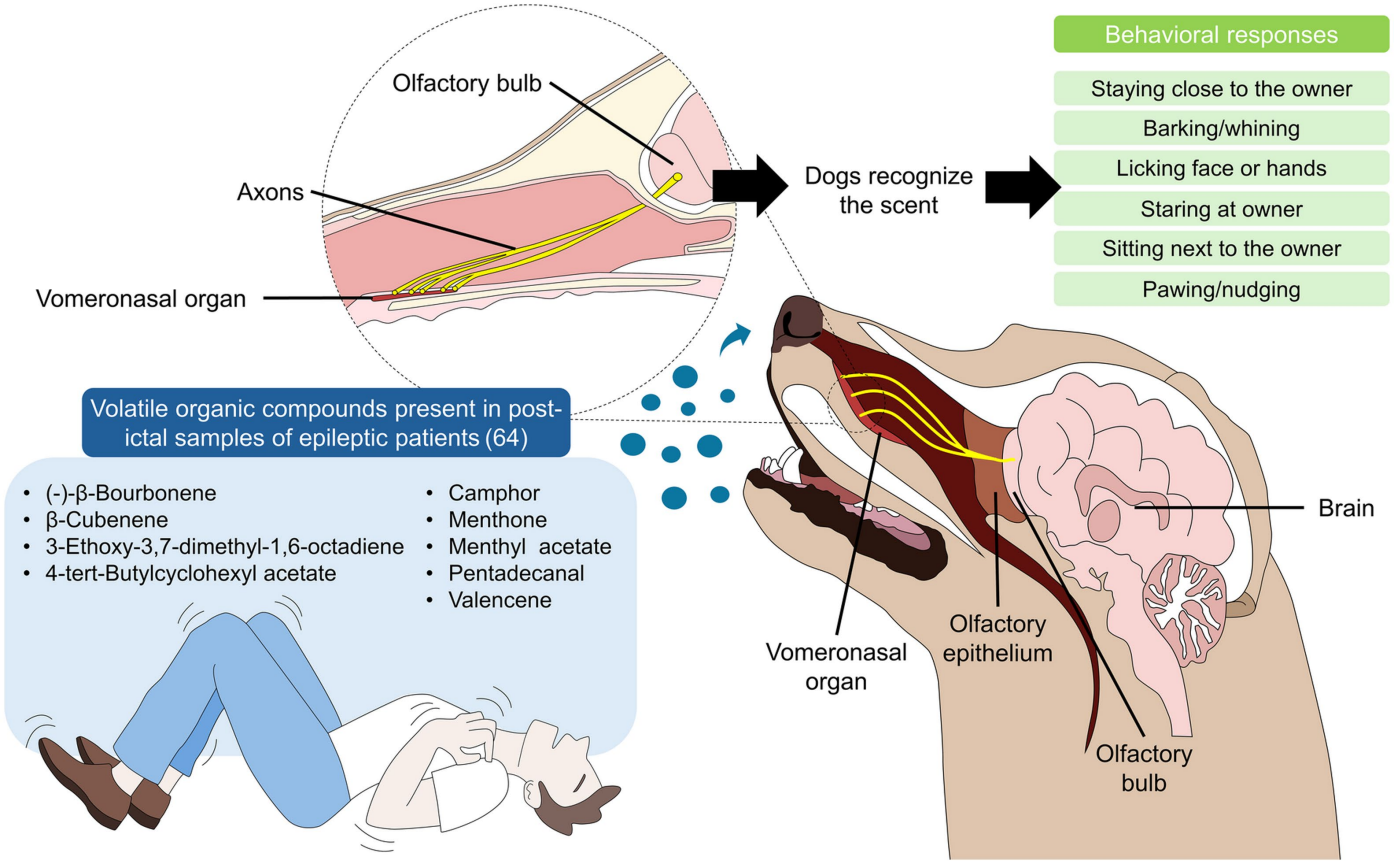
The “seizure scent” and the role of dogs as alert animals. Studies have shown that immediately after a seizure, individuals secrete volatile organic compounds (listed in the blue square at the left side of the image). Dogs recognize these compounds due to their keen olfactory ability. Olfactory signaling from the vomeronasal organ reaches the olfactory bulb via its axons, which subsequently project to the brain. Dogs that recognize this scent (seizure-responsive dogs) exhibit a wide range of behavioral modifications aimed at alerting the owner/patient or their caregivers (listed in the green squares on the right).

Dogs can alert and behaviorally respond to seizures by detecting a combination of VOCs produced in the immediate postictal period, which creates a unique scent. In this regard, Davis et al. (74) compared hand odor, saliva, and breath samples from epileptic and non-epileptic patients and identified at least 9 VOCs as biomarkers of the human scent associated with epileptic seizures (Figure 4) (70, 74). Menthone was considered a potential biomarker for epileptic activity as it was the only compound extracted from the three sample types, and exclusively from epileptic patients. Moreover, in canine trials evaluating whether dogs can alert to menthone, the authors found that 4/4 dogs imprinted on it (74).

Trained dogs (SRD) also respond to the general odor associated with epileptic seizure exhibited specific response behaviors (e.g., approaching and standing above the can with the steel test) within five minutes of exposure. These dogs recognized the seizure odor with a sensitivity ranging from 67–100% and a specificity between 95–100%, respectively (113). Similarly, Maa et al. (81) investigated whether trained dogs (Golden, Golden Doodle, and Labrador) were able to recognize the unique sweat scent of epileptic patients. Their results showed that dogs correctly distinguished 93.7% of ictal and interictal sweat samples. Furthermore, 78.7% of the dogs identified the scent prior to seizure onset, with a positive probability of 82.2% and an average warning time (from alert to seizure onset) of 68.2 min.

Kirton et al. (111) reported that the most common alert behaviors in SRDs were barking/whining (95%), followed by licking (50%), and close physical contact (32%). No false-positive responses were found. Catala et al. (114) reported behavioral changes in SADs, indicating that 30.6% of the dogs exhibited seizure-alert behaviors, including staying close to the patient (54.5%), staring (40.9%), whining (36.4%), and pacing (36.4%). Moreover, according to the Monash Canine Personality Questionnaire (MSPQ-R, which evaluates dogs’ personality), SADs have high scores for “motivation” and “training focus,” while according to the Monash Dog-Owner Relationship scale (MDORS, which assesses human–dog interactions), “emotional closeness” is also a relevant trait for SADs. Catala et al. (114) also mentioned that 45.5% of trained dogs adopted alert behaviors from the first day after exposure to a seizure. In comparison, 18.2, 13.6, and 22.7% exhibited alert behavior within the first week, 1 month, or after a few months, respectively.

The behaviors of SADs and untrained dogs has been reported, finding that the most frequent alerting behaviors among SADs were standing close to the patient and licking the patient’s face and wrists (70%) (108). Other behaviors included sitting nearby (30%) and pawing (20%). In contrast, untrained dogs most commonly exhibited staying close (62%), licking (48%), sitting next to the owner (41%), staring (40%), and vocalizing (32%) (e.g., barking). A notable difference in behavior between SADs and non-alerting dogs was that 57–51% of SADs remained close to the owner during and after seizures. In contrast, only 16% of non-alerting dogs performed this behavior. Powell et al. (112) also reported changes in behavior in untrained dogs (68.40% pedigree and 31.60% mixed breeds) when exposed to seizure phases. The authors evaluated time for eye contact (TEC), time near the owner (TNO), and time pressing close to the owner (including pawing/nudging) (TPC) during the ictal and post-ictal phases. They found that 70.8% of dogs-maintained eye contact with the owner, while 64.6 and 60.0% presented TNO and TPC, respectively. Additionally, TEC and TNO had significant longer durations during the post-ictal phase (median 53 s and 8 s, respectively), whereas TPC was significantly higher during the ictal phase (6 s).

In children, Kirton (18) found that 80% of SADs and SRDs presented alerting behaviors with a median accuracy of 80% and no false alerts. Reported behaviors included sitting on the person to prevent them from standing, or pushing the person away from stairs, and licking their feet. Although dogs have been proposed as companion capable of identifying seizure onset, Ortiz and Liporace (115) observed that both trained and untrained dogs’ performed poorly in clinical environments, such as epilepsy care units when compared to continuous electroencephalography. For example, in one patient with eight seizures, the SAD alerted only once, standing and staring at their owner for two seconds before the seizure. In another patient, the dog alerted seven minutes before an event, but the EEG revealed it was a non-epileptic seizure. During other episodes, the dog was not near the owner and therefore unable to respond. The authors concluded that environmental factors might have a substantial influence on the dog’s ability to alert prior to seizure onset.

Regardless of the environment, most studies report an increase in the quality of life (QoL) of humans and a reduction in the seizure frequency when living with a SAD or SRD (32). For example, 22 patients ranging from 12 to 66 years old, reported a significant increase in the QoL (83%) when living with a SRS, particularly an improvement in aspects such as significantly reduced depression (100%), as well as increased safety/security perception (87%), interpersonal relationships with strangers (82%), independence (87%), and self-confidence (77%) (111). In the same study, 45% of patients reported a significant decrease in the frequency and severity of the seizures when interacting with alerting dogs. Similarly, Kirton et al. (18) assessed the QoL of children living with SADs using the Impact of Pediatric Epilepsy Scale, where scores near zero represent worse QoL (116). The authors found that QoL scores were significantly higher in families living with a dog than in those without SADs (4.02 *versus* 3.46, respectively), and that children with dogs (SRD-SAD) showing alerting behaviors had the highest QoL scores (4.35 vs. 3.76). Of 48 families living with a dog, 42% reported seizure-related alert behaviors, including licking the face (59%), decreased motor activity (55%), “protective” behavior (50%), and whimpering (36%). The median anticipation time was 2.5 min with a sensitivity of 80%.

SADs have also shown an effect on the frequency of tonic–clonic seizures, as mentioned by Strong et al. (117), who monitored seizure frequency in patients with SADs during 48 weeks. When comparing baseline seizure frequency with the last weeks of evaluation, seizure frequency significantly reduced by 43%. Monthly tonic–clonic seizure frequency significantly reduced from 6.3–45.6 to a mean of 9.7 (1.7–37) and dropped further to 8.8 (1.7–30) after 48 weeks. Although no statistical analysis was performed, Strong et al. (82) reported a reduced frequency of human seizures after training dogs to perform alerting behaviors. Trained dogs (e.g., Labrador retrievers, Jack Russell, border collie, miniature schnauzer, and collie) successfully learned to bark, jump up, or paw within 15 to 45 min before the beginning of a tonic–clonic seizure.

To date, only hospital settings have technology that continuously monitors and alerts for seizure activity. Therefore, SADs and SRDs can be considered alternatives in domestic settings due to their capability to alert and show specific behaviors before seizure onset (Supplementary Table 1).

## 5 Animal-assisted therapy in hospitals

Hospitals are overstimulating environments that often lead to increased stress levels, decreased social interaction, and lifestyle impacts on patients (4, 118). In hospital settings, patients face physical, psychological, behavioral, social, and emotional challenges, which may include a combination of loss of control, sleep disturbances, malnutrition, restricted mobility, and pain (119, 120). This care focuses on alleviating some symptoms and stress, intending to improve QoL. Among this type of care, alternative methods to promote health have included AAI (121–123).

### 5.1 Recovering from heart failure and other ailments

The most prevalent cardiac condition in adults is heart failure, with more than 1.5 million cases diagnosed annually (124). In pediatric populations, the worldwide incidence of cardiac failure ranges from 0.97 to7.4 per 100,000, with approximately 11,000 and 14,000 children requiring hospitalization each year (125). In these cases, AAIs are used to mitigate the adverse effects of hospitalization, including distress, anxiety, and boredom (126). Beyond psychosocial support, several studies have noted the physiological benefits of AAIs. Walden et al. (127), for example, evaluated the impact of AAIs with dogs on physiological stability in hospitalized pediatric heart transplant patients (aged 6–19 years) diagnosed with congenital or acquired heart disease. The intervention consisted of activities such as walking, petting, and grooming the dogs over 1 week with therapy sessions averaging 17.2 min. Blood pressure and respiratory rate were recorded before and after each session. Results indicated that both systolic and diastolic blood pressure were significantly reduced following the dog assisted sessions (120–122 and 68 mmHg, respectively) compared with the non-AAIs group (126 mmHg). Lowering or maintaining a stable blood pressure is essential for individuals with cardiac disease, as it reduces vascular strain and contributes to improve cardiac function (128).

Benefits due to dog-assisted interventions were also reported in adult patients with critical heart failure. Cole et al. (2) assessed hemodynamic and neurohormonal parameters using a catheter in the pulmonary artery to measure blood pressure, pulmonary wedge pressure, and catecholamine levels (epinephrine and norepinephrine). Anxiety was assessed using the Spielberger State Trait Anxiety Index. Patients in the intervention group received a 12-min visit from a therapy dog team, while a control group did not. Monitoring was performed at baseline, during, and after the intervention. Significant decrease in blood pressure was observed in the intervention group both during (−4.32 mmHg) and after (−5.78 mmHg). Similarly, pulmonary capillary wedge pressure decreased during (−2.74 mmHg) and after (−4.31 mmHg) the intervention. Catecholamine concentrations were also significantly lower in the intervention group compared with the control group, with lower concentrations of epinephrine and norepinephrine during (−15.86 and −232.36 pg./mL, respectively) and after (−17.54 and −240.14 pg./mL, respectively). Additionally, the intervention group had a significantly lower total anxiety score (−9.13 units). These findings demonstrate that AAIs can improve cardiopulmonary pressures, modulate neurohormonal activity, and alleviate anxiety in hospitalized patients with heart failure.

### 5.2 Patients with cancer

A cancer diagnosis is frequently associated with distress. For example, Kurdistan et al. (129) reported average percentages of 33 and 27% for symptoms associated with depression and anxiety, respectively. The scores showed that the manifestations of anxiety were affected by chemotherapy cycles and the duration of the disease. Furthermore, a cancer diagnosis during childhood and adolescence can be detrimental to both emotional and cognitive development due to the immediate and extreme lifestyle changes, together with social impairments (130, 131). Psychologically, these patients experience fears about changes in their body image, loss of independence, and may feel hopeless and, of course, consider and confront their own mortality (132, 133).

Several studies have suggested the use of complementary medicine approaches, such as animal-facilitated therapy (AFT) and animal-assisted therapy (AAT) (also referred to as pet therapy). AAT is a goal-oriented treatment delivered with specific therapeutic objectives (3). In contrast, AFT is often used when referring to less structured interaction with animals focused on general well-being, instead of a clinical goal (134). These AAIs reduce distress in intensive care settings (135) or during hospitalization (136, 137). For example, in children (8 years), Silva and Osório (20) implemented an AAT program consisting of three sessions of 30 min using a retriever and a golden retriever dog. Self-assessment scales were used to evaluate pain and stress in children (138, 139), and anxiety, mental confusion, and tension in their caregivers (140, 141). Results showed that pet therapy significantly decreased pain perception (0.41 ± 1.01 pre-AAT vs. 0.08 ± 0.40 post-AAT on a scale from 0 to 10, *p* = 0.046) and stress levels (38.29 ± 17.75 pre-AAT vs. 30.25 ± 14.75 post-AAT on a scale from 0 to 140 points, *p* = 0.005) in children. Similarly, caregivers demonstrated reductions in anxiety, mental confusion, and tension levels decreased (to 26.04 ± 11.85, 1.71 ± 2.24, and 2.21 ± 2.06, respectively). These improvements in psychological parameters are attributed to the benefits of the human-animal relationship, potentially mediated by the release of oxytocin, endorphins, and serotonin, and to a reduction in the basal level of cortisol. These neurochemical changes contribute to reduce the perception of pain, anxiety, and stress, fostering sensations of pleasure and relaxation in children undergoing cancer treatment (142, 143).

The therapeutic effects of AAT have also been documented in adult oncological patients (average of 64.5 years) during weekly chemotherapy sessions (4). Psychological outcomes were assessed using the Anxiety, Depression, Somatic Symptoms, and Hostility test, alongside physiological variables including heart rate, blood pressure, and arterial oxygen saturation before and after the chemotherapy session. Results showed a significant reduction in anxiety levels (1.84 vs. 0.48), depression (1.04 vs. 0.70), and aggression (1.11 vs. 0.51), before vs. after the intervention, and no changes in somatic symptoms in the pet therapy group. Regarding physiological parameters, heart rate after interaction with the animal decreased significantly from 76.3 to 69.9 bpm, while oxygen saturation increased significantly from 97.47 to 98.04%. These findings highlight the emotional benefits of AAIs, while the increase in oxygen saturation may be linked to the interaction and physical activity associated with the animal interaction.

Aiba et al. (144) also conducted a pilot trial of AAT in a 79-year-old male patient hospitalized with colon cancer, lumbar abscess, diabetes mellitus, and a history of stroke with aggravated communication difficulties and requiring full tube feeding. Across four sessions, the dog was encouraged to interact with the patient. Progressive improvements in communication were observed: on day one, the patient was able to pronounce the word “dog”; in session two, the patient uttered two words and moved his arm to touch the dog; and by sessions three and four, the patient was able to verbally communicate with the caregiver by creating sentences and moving his limbs to call the dog. These results suggest a multifaceted effect whereby AAT motivates the rehabilitation of both physical and speech disorders and, while improving communication skills in the medical setting. Overall, animal-assisted therapy has been shown to provide complementary benefits to conventional medical treatment for transplant patients and individuals hospitalized with heart failure, improving physiological parameters and enhancing the quality of life of both patients and their families or caregivers.

## 6 Animal-assisted therapy for mental health

As a complementary strategy for the treatment of mental health disorders, AAT has been implemented with dogs and horses, aiming to reduce anxiety, depression, and post-traumatic stress by modulating physiological responses such as oxytocin release and cortisol reduction (5, 9). Studies have shown that AAT promotes motivation, empathy, and social skills, making it an effective and accessible resource for integrative psychotherapeutic interventions, as discussed below.

### 6.1 Schizophrenia and related disorders

Schizophrenia, a severe mental disorder with a worldwide incidence of 0.4%, is characterized by delusions, hallucinations, and cognitive dysfunction (145). In these patients, AAT has been shown to provide benefits, together with pharmacological interventions, in reducing negative symptoms such as apathy (anhedonia and alogia) and social withdrawal (146–148). This was found in a 6-month randomized clinical trial with patients diagnosed with chronic schizophrenia (average age of 47.8 years), where therapy dogs were part of the treatment for 6 months (146). The treatment included handling the dog, as well as walking, training, and playing with the animals weekly for 1 h. The Positive and Negative Syndrome Scale (PANSS) (149), and QoL scales such as EQ-5D were used (150), alongside salivary samples to determine cortisol and alpha-amylase levels as psychosocial biomarkers of stress. The authors found that interaction with therapy dogs significantly decreased PANSS scores (a decrease of up to 11.7 points, *p* < 0.05), where values near 30 represent no symptoms, and near 210 represent extremely severe symptoms. In contrast with no changes in the PANSS score in the control group. In addition, cortisol levels decreased significantly (by 0.50 μg/dL) after the session, suggesting modulation of physiological stress (146). A possible explanation can be found in the neurobiology of positive human-animal interactions, which induce pleasure and release of neurotransmitters such as endorphins, oxytocin, and dopamine. This neurochemical activity attenuates the activation of the hypothalamic–pituitary–adrenal axis pathway and consequently reduces cortisol release (5).

Similar results were observed in a 12-week randomized trial with 40 middle-aged and older adults (around 40 years old), in which AAT sessions with therapy dogs (breeds such as Labrador, Corgi, Maltese, and Shiba Inu) were provided weekly (151). Dog interaction consisted of physical (handling, feeding, grooming), cognitive (training and orienting the dog), social (talking to the dog), and sensory (touching, playing) activities. The results showed that patients in the canine treatment group had significantly greater increases in lower extremity strength (from 8.81 to 13.0) on the Chair Stand Test. Additionally, significant increases in the Assessment of Communication and Interaction Skills score were also recorded (71.50 to 75), with no differences in global cognition or final mobility. These findings reflect improved mental states in patients with severe cognitive dysfunction. In a geriatric population with schizophrenia (mean age 79.1 years), a closed psychogeriatric ward incorporated dogs and cats as “modeling companions” for 12 months (152). Individuals interacted with the animals by petting, feeding, grooming, and bathing them. Using the Scale for Social Adaptive Functioning Evaluation (153), the authors reported significant decreases in the social functioning subscale (from 42.6 to 27.6, with higher scores representing more severe impairment, *p* = 0.003), reflecting improvements in mobility, interpersonal contact, communication, daily living activities (personal hygiene), and independent self-care.

A systematic review conducted in 2019 (22) concluded that, despite the limitations posed by small sample sizes and variable risk of bias across clinical trials, AAT is generally associated with a significant reduction in negative symptoms such as anhedonia (lack of pleasure) and social withdrawal, and stimulates cognitive functioning, increasing the QoL of patients with mental health disorders. Moreover, AAT appears to stimulate cognitive functioning, thereby increasing the QoL of patients with mental health disorders. The review also highlighted that pet therapy may reduce the need for pharmacological therapy and, consequently, minimize associated side effects. In addition, AAT is also considered as a versatile intervention, applicable in different contexts including rehabilitation centers, hospitals, and nursing homes, among others (22).

### 6.2 Depression

Depression is a high-risk and high-incidence mental disorder in humans. According to the World Health Organization, at least 5% of adults suffer from depression worldwide, with a frequency of up to 50% in women (154). The symptoms include loss of pleasure, insomnia, fatigue, and feelings of worthlessness. The origin of depressive disorders is heterogeneous and composed of psychosocial, social, and even epigenetic factors such as physical pain, chronic stress, and abnormalities in brain circuitry (155). At least 700,000 deaths by suicide are recorded annually in depressed patients (154).

Animal-assisted therapy has shown a moderate but significant effect in reducing depressive symptoms, particularly in older adults (22, 156). For instance, a meta-analysis of randomized controlled trials (23) reported that most AAT uses dogs, cats, and birds, in therapy sessions ranging from 10 to 90 min, delivered over 6 weeks to 8 months. Patient-animal interactions mainly involve activities such as stroking and playing with the animal. Moreover, it was reported that AAT with dogs significantly reduced depressive symptoms. In contrast, a systematic review of the available evidence concluded that, although findings are generally favorable, the efficacy of AAT in managing depression in adults remains inconclusive, largely due to methodological heterogeneity and the limited number of high-quality studies (157).

In clinical settings, a recent controlled trial (from 2019 to 2020) compared a conventional rehabilitation program with one supplemented with dog therapy for 3 weeks in patients diagnosed with depression (average age of 60–70 years) (158). The conventional rehabilitation program included physiotherapy, occupational therapy, electrotherapy, and therapeutic massage. To estimate depression in patients, the authors used the Beck Depression Inventory scale before and after the intervention, with scores from 0 to 63 (159). Both groups significantly increased their QoL (0.926 vs. 0.903 Cohen’s, *p* < 0.001) and significantly decreased anxiety (0.984 vs. 0.857 Cohen’s, *p* < 0.001). However, only the standard program showed a significant decrease in depressive symptoms (a significant mean drop of up to 0.121 post-intervention), while the AAT group showed similar scores on indicators such as general well-being and pain. Despite this unfavorable result, other research (24) has shown a positive effect in elderly patients (≥65 years), significantly increasing their verbal interactions (<2 h per day) and positive emotional responses as measured by the Geriatric Depression Scale (24).

A limited number of studies have evaluated the impact of AAT on functional brain connectivity using magnetic resonance imaging. Kang et al. (160) evaluated the effect of equine-assisted activities and therapies (EAAT) on brain connectivity of 15 adolescents with attention-deficit hyperactivity disorder and severe depressive symptoms (11.7 points according to the Child Depression Inventory scale, K-CDI, on a scale from 0 to 60) (161). EAAT consisted of two 60-min sessions over 7 days, during in which the adolescent interacted with the horse through activities such as caring it, feeding it, brushing it, and therapeutic riding. Neuroimaging showed a significant increase in brain functional connectivity, particularly in the left and right amygdala, left parahippocampal gyrus, left medial frontal gyrus, left inferior frontal gyrus, left caudate, and right claustrum after EAAT. Moreover, a significant decrease in K-CDI scores (from 11.7 to 6.5, *p* < 0.01) was reported. Neuroimaging studies in animal models and humans with depressive disorders have shown deficient neuronal connections between brain regions such as the dorsal and medial prefrontal cortices, insular lobe, orbitofrontal cortex, amygdala, hippocampus, cingulate cortex, and nucleus accumbens, due to metabolic slowing accompanied by reduced blood flow in these brain regions (155). Thus, this shows a modification in affective neural networks similar to that observed in healthy adolescents and suggests considerable therapeutic efficacy.

In summary, AAT, especially using dogs, has potential benefits in reducing depressive symptoms in older patients, although its efficacy is more modest than that of conventional therapies. The evidence suggests positive effects on QoL, anxiety, and perceptions of control. However, understanding still needs to be strengthened through better-designed trials with larger sample sizes, blinding, precise intervention dosage, and long-term follow-up. In addition, sophisticated study tools such as neuroimaging should be included to reinforce scientific findings.

### 6.3 Anxiety

Anxiety disorders include a range of presentations such as panic disorder, generalized anxiety disorder, social anxiety disorder, and separation anxiety disorder, and affect 33.7% of the world’s population (162). Pathological anxiety can manifest through signs such as trembling, sweating, paleness, body ache, heart palpitation, and syncope. It has a chronic course and can be aggravated by social, economic, or environmental changes (162).

AAT has shown favorable, short-term effects on anxiety in various clinical and community settings (163, 164). For example, a recent randomized clinical trial in a pediatric emergency department (mean patient age 10.9 years) evaluated the effect of a brief (10-min) interaction with a therapy dog (25). The authors assessed anxiety scores through the 0- to 10-point FACEs anxiety scale (165), as well as salivary cortisol. AAT significantly reduced FACES scores after the interaction, from 5.4 to 3.0, where higher scores mean greater anxiety (*p* = 0.02). Salivary cortisol levels also decreased by −0.04 μg/dL in children. Similarly, Kline et al. (26) assessed the benefits of a 15-min visit with a therapy dog in an emergency department. Adult patients in the AAT group interacted freely with the dog. After the interaction, their anxiety levels were evaluated using the FACES. The findings showed that the AAT group had average post-session scores of 6 (moderate worry), in contrast to the standard treatment group (not AAT), which obtained scores of 8 (feeling really bad or the highest level of distress/pain). The patients also presented behavioral changes associated with improved mood, including changes in verbal expression, laughter, and relaxation. In particular, it is estimated that 81% of patients admitted to emergency care rooms have reported acute stress in the last 3 months, and 50% of them had anxiety episodes (26). Therefore, the application of AAT represents a complementary approach that improves the patients’ experience in the emergency care settings, and that facilitates the non-pharmacological management of negative emotions such as anxiety.

In children (6–17 years), where approximately 20% of the population is diagnosed with anxiety disorders (166), AAT, consisting of equine-assisted therapeutic riding during 10 weekly sessions, has shown positive effects (166). Anxiety symptoms were assessed through the Screen for Child Anxiety Related Disorders scale, with a total possible score of 82 (167). AAT significantly reduced anxiety, particularly in patients above 9 years (from 54 to −13.80, *p* = 0.042), while self-efficacy for adaptive equestrian training increased, supporting the value of integrating Cognitive Behavioral Therapy principles with animal interaction in the at-risk population. Likewise, in university students who belong to the at-risk population, and where stress and anxiety are prevalent, a systematic review and meta-analysis of randomized trials found that canine-assisted therapy significantly reduced anxiety and stress (8). However, canine-assisted therapy has variation in methodology and short-term effects (8). Environmental factors pose challenges to mental health, such as academic workload pressure, inadequate sleep, among others, have contributed to the persistence of psychological distress in 73% of students in the United States (8, 168). Pendry et al. (7) reported complementary physiological findings, demonstrating that brief animal visitation programs of 10-min on university campuses significantly decreased salivary cortisol levels by an average of 1 μg/dL on average. This finding suggests attenuation of the hypothalamic–pituitary–adrenal axis activity, occurring in parallel with reduction of discomfort. These results are particularly relevant given the elevated suicide rate among students experiencing high levels of anxiety (8, 169).

However, a recent review highlights the need for greater rigor in methodological factors including larger sample sizes, blinding when feasible, standardization of the “dose” of AAT (frequency/duration), and follow-up assessments to determine the persistence of effect (8). In summary, AAT, particularly with dogs, and to a lesser extent structured equine intervention, represents a promising adjunctive strategy for reducing anxiety in both emergency settings and educational environments.

## 7 Animal assisted intervention for the elderly

Dogs can have a significant positive impact on human lives, especially among older adults, who are likely to experience health problems such as visual and hearing impairments (170). The benefits can also be reflected in the mental, physical, and social health (171, 172). For example, when retiring, older people might experience solitude due to a lack of social interaction, which can increase emotional burden (170). In this context, owning a dog or participating in AAT can help structure a daily routine, since the responsibilities associated with caring for the dog, such as feeding schedules and walks, potentially offer seniors a new sense of purpose and commitment (173). Yabroff et al. (174) reported that older people who own a pet walk up to 18.9 min more per week than those without pets. In addition, human-animal interaction has a direct effect on mental health by reducing anxiety and loneliness, and facilitating memory (175). This is relevant since older people are at higher risk of suffering mental deterioration, and one of the leading causes of disability and mortality in this age category is dementia, so multidisciplinary strategies are needed to reduce its prevalence (176).

### 7.1 Senile dementia

Alzheimer’s disease, also called “senile dementia,” is a progressive neurological disorder characterized by a impaired memory, cognitive function, and difficulty in performing everyday tasks (177). To date, there is no cure for senile dementia, and non-pharmaceutical interventions focus on preserving the patient’s QoL. In this sense, AAIs with dogs have been proposed as coadjutant, as mentioned by Moretti et al. (178), who evaluated the effects of dog therapy on the cognitive function of 21 dementia patients with a mean age of 84.7 years. In the intervention group, participants were allowed to hold, stroke, walk, talk, and play with four dogs for 90 min once a week for 6 weeks. The patients were evaluated using the Mini-Mental State Examination (MMSE, a scale from 0 to 30, where scores near 30 represent no impairment, and those close to o severe impairment) and the Geriatric Depression Scale (GDS, scoring from 0 to 15, with higher scores interpreted as severe depression) before and after the intervention (179, 180). The results showed that the dog therapy group significantly reduced their geriatric depression symptoms by 50% (*p* = 0.013) and significantly increased their average MMSE score by 4.5 points (*p* = 0.060), which is twice as much as the control group (178). Likewise, Menna et al. (6) evaluated the effectiveness of AAT in 50 patients with Alzheimer’s disease, with an average age of 75 years. The results concluded that the mean GDS score of the AAT group decreased significantly from 11.5 to 9.5. On the other hand, the total MMSE score decreased significantly from 21.5 to 20.5, indicating that TAA interactions are applicable and effective in stimulating cognition and improving mood in patients with dementia (6).

### 7.2 Dog-therapy for the elderly housed in nursing homes

Considering that older adults experience declines in their general faculties, poor health and functional limitations, these factors often compromise their ability to continue living in their homes. Loss of independence and the need for assistance are frequent reasons for moving to an assisted living facility. In many cases, institutionalization of older adults leads to loneliness, isolation, and loss of interest in various activities, which can, in turn, lead to the appearance of depressive symptoms (181).

The use of AAI programs has been promoted in nursing homes, and visiting dogs are increasingly valued as a novel option for promoting the psychological well-being of older adults in institutions, due to their ability to foster communication, reduce loneliness, and alleviate depressive symptoms (182). One way to measure the impact of dog visits in nursing homes is to assess residents’ psychological well-being using psychiatric scales that analyze aspects such as depression and cognitive functioning (183). For example, the GDS was used to evaluate the effect of AAT sessions over 10 weeks (30-min sessions, weekly) in geriatric patients. After the intervention, scores on the GDS test decreased by an average of 33.5%, indicating a significant decrease in patients’ depression (*p* = 0.0004) (24). It has also been shown that, with increasing age, the circadian rhythm and sleep quality worsen, so measuring sleep quality is an objective measure of well-being in older people. For instance, the effect of therapies involving a dog, a robot seal, and a stuffed cat was evaluated in older adults (183). The sessions consisted of 12 biweekly visits of 10 min each. At the end of the experiment, it was observed that sleep duration per week significantly increased by up to 610 ± 127 min in the third week when accompanied by a dog, compared to when they were accompanied by the robot seal (498 ± 146 min) or the stuffed cat (540 ± 163 min). Although a transient effect on sleep duration was observed (increasing in the third week but disappearing by the sixth), more experiments across different settings are required to fully understand the benefits that AAI have for this population.

## 8 Autism spectrum disorder

In 2018, the prevalence of autism spectrum disorder (ASD) increased from 0.67 to 1.85% (184–186). ASD varies from individual to individual (187). However, it includes various neurodevelopmental disorders characterized mainly by social and communication deficits (187, 188). These are often observed as difficulty in understanding and expressing emotions, limited speech, difficulty navigating social interactions, and repetitive or restricted behaviors, among others (189). Several studies have shown that children and adults with ASD benefit from physical interaction with animals (e.g., dogs and horses) through pet ownership or AAT, considered a socio-communicative intervention that increases the well-being of people with ASD (29, 190, 191).

**Figure 5:**
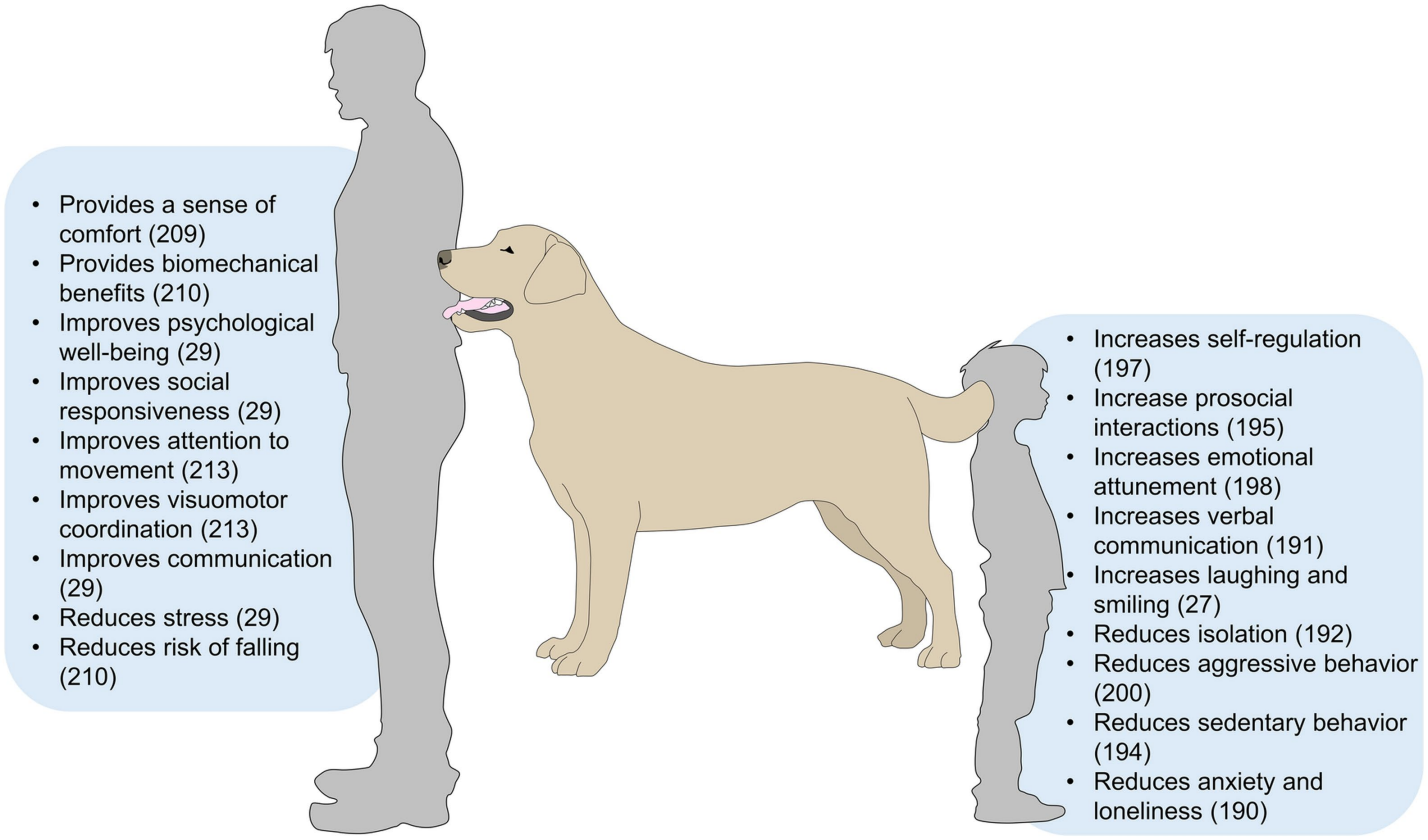
Effects of animal-assisted interventions in individuals with autism spectrum disorder.

Animals are nonverbal and communicate through behavior and body posture. Thus, they can serve as a communication bridge between non-human animals and humans, reducing the stress that individuals with ASD might experience when interacting with others (29). AAT has shown improvements for social skills and functioning in individuals with ASD (30, 191). For example, a systematic review concluded that 79% of studies evaluating the effect of AAT on ASD focused on language and communication outcomes, and 75% of these studies reported significant improvements in both aspects (Figure 5) (29, 187, 192–194).

### 8.1 Dog-assisted therapy in individuals with autism spectrum disorder

In children and adolescents, AAT has been widely used as a complementary treatment for ASD because animals serve as transitional objects with which children can form bonds and then extend those bonds to other humans, increasing the likelihood of prosocial interactions (27, 195). In particular, dogs use nonverbal communication and exhibit intentional behaviors, which tend to be easier for children with ASD to comprehend (191). Children can develop a sense of connection with the animals by learning to gently pet and talk to them, which, in consequence, reduces anxiety and loneliness (190). For example, Redefer and Goodman (192) evaluated the effect that 18 sessions of pet-assisted therapy had on the social behavior of autistic children (5 to 10 years old). At the beginning of the sessions, the children displayed behaviors often associated with ASD such as social withdrawal and unusual or absent language. However, after encouraging interactions between children and dogs (e.g., feeding, grooming, ball-throwing), social interaction significantly increased from 2.8 ± 1.6 to 9.0 ± 1.7, while isolation behavior significantly decreased from 17.2 ± 1.9 to 10.5 ± 1.8.

Limited prosocial initiation and engagement, as well as restricted emotional responsiveness, are characteristics of social impairment in children with ASD. In this sense, Griffioen et al. (196) reported changes in behavioral synchrony (defined as “an observable pattern of dyadic interaction that is mutually regulated, reciprocal, and harmonious”) during dog therapy. Children (12–18 years old) diagnosed with ASD, pervasive developmental disorder-not otherwise specified, multiple complex developmental disorder, and intellectual disability were included in six weekly sessions of interaction with dogs (Labrador and Labradoodle breeds). As part of the session, children-animal interaction encouraged small exercises and giving certain commands to the dog. The authors used the Child Behavior Checklist (CBCL) to evaluate the effect. After the AAT, children with ASD improved their proportion of synchrony from an average of 0.02 to 0.09. Moreover, CBCL for internal problems significantly decreased from 12.30 to 9.80, and the total CBCL score from 56.90 to 50.30. These results indicate a significant reduction in behavioral and emotional problems in individuals with ASD (193), which is a desired outcome to improve the social skills of people with ASD.

Behavioral benefits due to AAT were also reported by London et al. (197) in children from 4 to 19 years diagnosed with ASD. Before the therapy started, the most commonly reported symptoms in children were maladaptive behaviors (88.2%), routine dependency (88.2%), impaired socialization and regulation (82.4 and 70.5%, respectively), and sensory responses (64.7%). After dog therapy (consisting of brushing, feeding, walking, and playing with the dog), 100% of parents reported children’s engagement in dog-caring activities (brushing, feeding, walking, and playing with the dog). Parents reported that the children’s engagement was also present in their daily routine. Additionally, 94.1% of parents reported enjoyment during the sessions, 70.6% reported that the dog helped the child to self-regulate, and 64.7% indicated that the dog’s presence significantly increased the ease of the children to communicate with the therapist. A significant increase in emotional attunement and regulation was also reported when comparing the effect of AAT with Labrador and Poodle dog breeds (198). Children (7–17 years) diagnosed with ASD requiring substantial support and having different levels of intellectual disability (e.g., IQ or intelligence quotient as low as 50–69) were exposed to AAT for 4–6 weeks. The child and dog had to complete an obstacle course together. Although no significant differences were found for social confidence and social motivation, significant increases were observed in emotional attunement (from 2.94 ± 0.48 to 3.09 ± 0.29) and emotional regulation (2.83 ± 0.30 to 3.18 ± 0.19). Moreover, another study reported a significant increase in light physical activity (by 13%) and a significant reduction in sedentary behaviors due to AAT (22%) (194). These findings are relevant since children with ASD tend to have lower physical activity levels than children and youth without autism (199).

Another desirable outcome of AAT in children with ASD is the increase in the frequency of positive behaviors such as laughing and smiling. In this sense, Martin and Farnum (27) compared AAT with dogs (Clumber Spaniel, Newfoundland, and Border Collie/Yellow Lab cross) with providing a ball and a stuffed dog to children from 3 to 13 years old diagnosed with ASD and with developmental ages of 2.5 to 6.5. After 15 weeks of AAT, the authors evaluated behavioral and verbal responses. It was found that interactions with dogs significantly increased the frequency of laughing (0.57 ± 1.18) when compared to the ball or stuffed animal condition (0.41 ± 1.06 and 0.21 ± 0.64, respectively). Additionally, children talked about themselves for significantly longer times (0.75 ± 2.28) than in the stuffed-dog group (0.46 ± 1.62). Children also talked more to the dog (0.55 ± 1.21), and they were more likely to engage the therapist in talks about the dog (0.73 ± 1.27). Similarly, Silva et al. (200) published a case report of 12-year-old boy diagnosed with DSM-IV ASD, where therapy with a dog significantly increased the frequency and duration of positive behaviors such as smiling (5.00 ± 1.57 vs. control 0.67 ± 0.49 and 8.18 ± 3.57 s vs. control 2.34 ± 1.86 s) and positive physical contacting (21.83 ± 2.81 vs. control 11.83 ± 1.60). Aggressive behaviors in the child were also significantly less frequent with the presence of the dog.

Regarding social communication in children with ASD (ages 8–14), Becker et al. (28) incorporated dogs into social skills training (SST) for 12 weeks of weekly treatment. After the SST, the authors found that children in groups with dogs experienced significantly greater reductions in symptoms, as measured by the Interpersonal Problems subscale (201) (from 66 ± 16.7 to 53 ± 11.9, *p* < 0.01), where higher scores indicate greater distress. A reduction in the score of the Functional Problems subscale was also reported (from 66 ± 14.9 to 54 ± 9.5, on a scale where a higher score means more significant functional problems). The same children had significantly fewer social deficits and more social communication, accompanied by fewer repetitive behaviors than students in the group with no dogs. Likewise, in preschool autistic children, exposure to therapy dogs significantly increased the frequency of social initiations by greeting a social partner (2.33 ± 0.11, *p* < 0.05) (202). These results are similar to those reported by Fung et al. (191) in autistic children (aged 7–10) attending dog therapy. After 14 individual sessions, verbal social behavior increased significantly from a frequency of 6 to 12 (*p* = 0.043). The benefits that interaction with animals has on the communicative skills of people with ASD are crucial, particularly when animals elicit verbal responses from children, since approximately 25–30% of people with autism are nonverbal or minimally verbal (203).

Another benefit that has been reported in children with ASD due to AAT is the reduction in repetitive behaviors such as humming, clicking noises, hand-posturing, roaming, and jumping (189, 204). As Ang and MacDougall (189) mention, during AAT, children are more aware of the animal’s presence, which regulates their sensory stimulation and also might have a calming effect mediated by oxytocin release.

In adults with ASD, research mainly focuses on the effect that AAT has on psychological symptoms (e.g., depression and anxiety), social responsiveness and communication. An example is Wijker et al. (29) who carried out a10-week study in adults (18–60 years) diagnosed with ASD and having an IQ of 80 or above. Individuals participated in AAT sessions where free interaction with dogs was allowed (Labradors, Labrador crossbreeds, Golden retrievers, Poodles, and German Wirehaired Pointer dogs were used). The authors utilized the Perceived Stress Scale (scoring from 0 to 40, where higher numbers represent high perceived stress) (205) and the Social Responsiveness Scale for Adults (SRS-A, on a scale from 0 to 195, with values ≥ 76 representing severe impairment) (206) to assess stress-related outcomes following AAT. Results indicated that the mean scores for stress significantly decreased to approximately 20 points (*p* = 0.02), while the SRS-A score for impairments in social responsiveness decreased (from above 60 points). This results reveals an improvement in psychological well-being among adults resulting from human–animal interactions. Similarly, Wijker et al. (207) determined the effect of dog-assisted therapy (DAT) on communication skills and self-esteem of adults with ASD and an IQ score above 80. During the sessions, the therapist encouraged the patient to be aware of the dog’s presence. After 10 weeks of therapy, the scores for the Rosenberg Self-Esteem Scale (a method used to measure self-reported self-esteem, a 4-point scale) (208) significantly increased for secure posture from 2.44 to 3.44 (*p* < 0.05). This might be related to the sense of comfort and decreased stress perception when interacting with animals, as reported when evaluating the autonomic and endocrine effects of DAT on adults with ASD without intellectual disability (209). Although no differences were found between sympathetic and parasympathetic activity (as measured by heart rate variability), blood cortisol levels decreased from 10.04 ± 3.14 to 8.74 ± 3.14 nmoL/L after the intervention with dogs. These findings suggest that AAT reduces acute stress.

Apart from behavioral and emotional benefits, AAT for individuals with ASD offers biomechanical benefits, as reported by Fernández-Sánchez et al. (210) in adults (36 to 57 years) participating in DAT sessions for 10 weeks. Biomechanical enhancements, such as improvement in the gait [Tinetti test (211) from 7.33 to 8.00, on a scale of 12 points], balance (from 10.67 to 11.83, on a scale of 16 pts), and risk of falling (from 5 to 2) were recorded after DAT (*p* < 0.05). Gómez-Calcerrada et al. (212) reported similar findings in adults older than 40 years old, diagnosed with ASD, and with enough functional capacity to walk independently. These adults participated in AAT with a Labrador retriever. The sessions promoted human-animal interaction by climbing stairs, walking, and moving through an obstacle course. Results showed improvements in gait and posture, which directly correlate with the ability of ASD individuals to perform daily living activities. The same results were reported by Scorzato et al. (213) in autistic adults with severe to profound intellectual disability who attended DAT over 20 weeks. Patients showed significant improvements in cognitive domains, including attention to movement (scores from 6.50 to 15.50), visuomotor coordination (from 10.48 to 14.67, *p* < 0.0001), exploration play (from 15.55 to 24.12, *p* < 0.0001), and motor imitation (from 18.71 to 25.71, *p* = 0.024), as well as in some social skills (from 57.4 to 67.1, *p* < 0.0001). Fine motor skills are necessary for self-care and social activities. Thus, improving these skills through DAT provides physical and social benefits for individuals with ASD.

### 8.2 Equine-assisted therapy in individuals with autism spectrum disorder

Horses are another species frequently incorporated into therapeutic programs for individuals with ASD (184). Therapeutic horseback riding (THR) is considered an effective form of AAT. Borgi et al. (214) investigated equine-assisted therapy (EAT) over a six-month period in children with ASD (aged 6–12 years) and IQ above 70. The intervention included grooming, hand walking, and horseback riding. Results indicated improvements in problem-solving tasks (planning time significantly decreased by 20.7 ± 6.6 s in individuals receiving EAT, *p* < 0.05). Moreover, socialization scores significantly increased to up to 0.72 ± 0.22 on the Vineland Adaptive Behavior Scale (*p* < 0.05), where the item scoring ranges between 0 and 2. Similarly, it has been reported that an EAT positively influenced communication skills and executive and motor function in children with ASD (aged 9–14 years) (215). Riding activities were used to stimulate both gross and fine motor control. According to the Social Responsiveness Scale (SRS) and Sensory Profile, improvements were observed in communication (18.3%), affective state (23.33%), social attention (33.5%), physical movements (34.1%), and dynamic motor skills (35.55%) (*p <* 0.01). Bass et al. (216) found in children (aged 5–10 years) with ASD, that THR sessions conducted over 12 weeks significantly increased the scores for sensory seeking (from 58.4 ± 10.6 to 62 ± 9) and sensory sensitivity (from 15.7 ± 3.6 to 17.2 ± 2.6) on the subscale SRS (216). In general, these findings suggest that equine-assisted interventions foster higher social motivation and decreased inattention among individuals with ASD.

In children and adolescents, Gabriels et al. (30) evaluated the effectiveness of EAT on self-regulation, socialization, and communication in children with ASD. Caregivers evaluated the child’s behavior before and after the intervention through the ABC-C scale (the Aberrant Behavior Checklist), a questionnaire that measures behavioral changes in children, where higher scores indicate more severe challenging behaviors (217). When compared to the control group, children in the EAT group had significant improvements, such as a reduction in irritability (by 6.3 ± 1.08) and hyperactivity scores (reduced by 7.5 ± 1.25 points) (*p* < 0.05). Social cognition significantly increased from 17.6 ± 5.55 to 20.3 ± 5.63, along with the total number of words (increased from 104.6 ± 58.45 to 116.7 ± 66.0) and use of new words (increased from 219.2 ± 132.19 to 253.7 ± 154.62) (*p* < 0.05). Likewise, Chen et al. (184) reported a positive influence of THR on autistic children (6–12 years). The Social Skills Improvement System Rating Scales score (218) and the Assessment of Basic Language and Learning Skills-Revised scores significantly increased from 44.68 ± 7.48 to 50.87 ± 6.47 and from 24.03 ± 3.38 to 33.84 ± 4.00, respectively, after 16 weeks of intervention (*p* < 0.05). Therefore, EAT programs can enhance the social and communication skills of people with ASD (219, 220) (Supplementary Table 2).

## 9 Animal welfare in animal-assisted interventions

Due to its therapeutic focus, AAIs have been proposed as a valuable and complementary approach for human well-being. However, it is important to consider that AAIs place cognitive demands on animals, making them susceptible to stressors. This underscores the ethical imperative for interventions that prioritize the impact on the health and well-being of participating animals (221).

The main risk to animal welfare is the development of acute or chronic stress, induced by unfamiliar environments, excessive or inappropriate physical contact, and the need to maintain high behavioral performance for prolonged periods. For example, Haubenhober et al. (222) reported significantly higher salivary cortisol concentrations (5.71 NmoL/L–1 vs. 2.59 NmoL/L–1 control days) in dogs after each therapeutic session (1 h–8 h). Furthermore, dogs attending a greater number of sessions quarterly (50) showed salivary cortisol levels of up to ~80 NmoL/L–1, in contrast to those attending ≤30 sessions (~40 NmoL/L–1). This is associated with an adaptive physiological response as a compensatory measure to a negative stressor. This can also be accompanied by behavioral manifestations such as panting, pupillary dilation, yawning, and whining, which reflect the animal’s negative internal state in response to the prolonged duration of the sessions (223). In addition, environmental conditions such as limited access to water and variations in ambient temperature can exacerbate the animal’s fatigue (224).

Furthermore, interaction with humans can be perceived as unpleasant by some dogs and in some contexts, due to factors such as age and familiarity with humans or specific human behaviors. For example, there is research on the benefits of AAT in fourteen students with severe disabilities, which was abruptly interrupted due to the dog exhibiting signs of stress (fatigue and frequent panting), leading to a deterioration in their health (225). Children who physically interact with an unknown or familiar dog may experience aggression from the dog; this is associated with discomfort due to low tolerance for physical intimacy (226). Physical interaction through petting with strangers triggers a significantly lower frequency (<1 in 60 s) of positive redirected behaviors such as sniffing/licking on the floor, playing with inanimate objects, digging, drinking, and visual scanning, in contrast to familiar persons, indicating an emotional state of motivational conflict on the part of the dog (227).

Based on scientific evidence, it is suggested that stress can be significant if AAIs are not properly managed, jeopardizing the animal’s emotional and physical welfare. To ensure a positive or at least neutral impact on the animal, ethical AAT programs have incorporated rigorous protocols and guidelines to reduce work-related stress and improve the welfare of therapy animals. For example, the International Association of Human-Animal Interaction Organizations (IAHAIO) has published guidelines for AAI (228). The Austrian Ministry of Labor, Social Affairs, and Consumer Protection regulates the certification of therapy dogs through the Federal Disability Act and specifies periodic health, temperament, and behavioral reviews (229). These guidelines support the importance of recognizing signs of distress (e.g., avoidance, growling, freezing, or biting) and providing environmental enrichment to increase social responsiveness in animals. Moreover, animals need to be able to retreat from overwhelming situations even during AAI (230).

The impact of AAT on animal welfare is not inherently negative, but it depends entirely on professional management and the recommended application of ethical guidelines. Only when the animal’s welfare, safety, and autonomy are considered as important as the human therapeutic benefit can the incorporation of animals into AAI be morally justified.

## 10 Conclusion

Animal-assisted therapy (AAT) has emerged as an innovative and effective therapeutic tool in hospital and clinical settings. The evidence reviewed demonstrates significant changes in physiological, hormonal, and psychological parameters in both adults and children, reinforcing its value as a complementary strategy in the care of patients with chronic diseases, heart failure, and cancer. Integrating AAT into hospital environments can contribute to a more humane, patient-centered model of care.

Likewise, MDDs also play a crucial role, serving as alert for conditions such as diabetes and epilepsy. Moreover, AAT has proven to be an effective adjuvant in mental health, with documented benefits for individuals experiencing anxiety, depression, and schizophrenia. These benefits range from reducing physiological stress to enhancing social skills and overall well-being.

In elderly population and individuals with ASD, interaction with animals has been shown to improve the QoL and promotes prosocial behaviors, particularly in those with compromised social functioning. While further research is needed to fully understand the role of animal companionship on several human health conditions, current evidence supports that the human-animal interaction through AAT and broader AAI provides substantial benefits to human health.
