# Supplementary material for: An overview of the benefits of animal-assisted interventions in medical and therapeutic contexts for human health: cognitive mechanisms, sensory perception and welfare considerations
Source: Front Vet Sci. 2026 Mar 20;13:1757427. doi: 10.3389/fvets.2026.1757427 (PMC13046555; doi:10.3389/fvets.2026.1757427)
Supplement: Supplementary file 1 [file Data_Sheet_1.pdf]

Supplementary table 1.

AAI type and disease	Evidence type	Species and breed	Age	Experience	Attention-seeking behaviors	Specificity or sensitivity	Reference
DADs	Anecdotal	1 Labrador Retriever Dog	N/S	Trained	Sniff out	N/S	Lippi et al. (17)
DADs	Anecdotal	212 domestic dogs (70.2% pedigree and 29.8% mongrel)	<6 months (1.4%)<br><br>-7 to12 months (7.1%)<br><br>-1 to 5 years (46.7%)<br><br>>5 years (44-8%)	No-trained	-Vocalizing<br><br>-Licking<br><br>-Nuzzling<br><br>-Jumping on them<br><br>-Staring into the face	65.1%	Wells et al. (90)
DADs	Empirical	6 dogs:<br><br>-Labrador retriever (2)<br><br>-Flat-coated retriever<br><br>-Siberian husky mix<br><br>-Spaniel mix<br><br>-German Shepherd	-9 to18 months	~6 months of training for hypo alert	-Smelling<br><br>-Touching<br><br>-Sitting	-Sensitivity 50.0–87.5%<br><br>-Specificity 50.0% to 89.6%.	Hardin et al. (84)

DADs	Empirical	27 dogs:<br><br>-Golden x Labrador Retriever<br><br>-Curly Coat x Labrador Retriever<br><br>-Bichon Frise<br><br>-Cocker Spaniels - Poodle<br><br>-Poodle cross<br><br>-Staffordshire<br><br>-Bull Terrier<br><br>-English Springer Spaniel<br><br>-Yorkshire Terrier	N/S	Trained	-Nuzzling<br><br>-Nudging<br><br>-Pawing<br><br>-Staring	-Mean sensitivity 70%	Rooney et al. (87)
DADs	Empirical	8 dogs:<br><br>-Labradors<br><br>-Labrador-Golden Retriever crosses<br><br>-Miniature Poodle<br><br>-Golden Retriever.	-12 to 72 months	Trained	-Fetch blood testing<br><br>-Stare at owner<br><br>-Nuzzle owner<br><br>-Mouth owner<br><br>-Paw owner	-Sensitivity to hypoglycemic (55.9%) and to hyperglycemic (36.5%)	Wilson et al. (86)

					-Lick owner<br>-Jump up on the owner		
MDDs (melanoma)	Case report	-Male Labrador	2-year-old	No-trained	-Sniffing a 2-cm mass on its owner's leg	N/S	Moser et al. (94)
MDDs (Sarcoma, carcinoma, and melanoma)	Empirical	6 pet dogs:<br>-mixed breed<br>-Weimaraner<br>-Beagles	~5.4 years	-Trained (completed 6 months of canine scent work training)	-Sit down in front of the samples (saliva)	-90% sensitivity<br>-98% specificity	Malone et al. (93)
MDDs (cell lung cancer)	Empirical experimental	3 Beagle	2-year-old	Trained (over 8 weeks)	-Sitting in front of the samples (serum)	-Sensitivity 96.7%<br>-Specificity of 97.5%	Junqueira et al. (97)
MDDs (lung cancer)	Empirical experimental	Golden Retriever	7-year-old	Trained (one year)	By pausing more than five seconds with its nose in the funnel of samples (urine and breath)	-Detection rate of urine samples (87.8%) and breath samples (78%)	Feil et al. (98)
MDDs (osteosarcoma)		2 Belgian Malinois dogs	1 and 7 years	Trained (16 months)	-Sit stare (cell culture medium and saliva)	- Sensitivities and specificities of 95%-100%	Ortal et al. (100)

SADs and SRDs	Anecdotal	160 pet dogs	N/S	Trained and untrained	-Licking the owner's hands and face<br><br>- Staying close to the owner<br><br>-Sitting<br><br>-Touching<br><br>- Vocalizing (barking, growling, whining)<br><br>-Jumping	-Detection rate trained dogs (63%) 30–60 min before its onset<br><br>-Detection rate of no-trained dogs (36%) 0–10 min before the onset	Martinez-Caja et al. (108)
SADs	Experimental	5 neutered dogs:<br><br>Golden/Labrador retriever mix<br><br>-Border collie mix<br><br>- Chesapeake bay retriever mix<br><br>- Dachshund - Australian shepherd mix	-From 2 to 5 years	Trained	-Dog standing above the scent (sweaty hands, forehead, and back of the neck)	- Sensitivity ranging from 67–100%<br><br>-Specificity between 95–100%	Catala et al. (113)

SADs and SRDs	Experimental	13 dogs:<br><br>-Golden<br><br>-Golden Doodle<br><br>-Labrador	N/S	Trained	- Touching of the trainer's LEFT hand (underarm sweat samples)	-Detection rate of 93.7% of ictal and interictal sweat samples	Maa et al. (81)
SRDs	Anecdotal	22 pet dogs:<br><br>- Labrador retrievers<br><br>-Standard poodle - Border terrier<br><br>-Toy poodle<br><br>-Cocker spaniel	~4.8 years	Trained (4 weeks)	Barking/whining<br><br>-Licking<br><br>-Close physical contact	Spontaneous alerting behavior developed in 59%	Kirton et al. (111)
SADs	Anecdotal	72 dogs:<br><br>-Golden Retriever<br><br>-Labrador Retriever<br><br>-English Cocker Spaniel<br><br>-Cavalier King Charles Spaniel<br><br>-Boxer<br><br>-Chihuahua	From <2 months to >1 year	Trained and untrained	-Staying very close<br><br>-Staring<br><br>-Whining<br><br>-Pacing around<br><br>-Going to another person<br><br>-Licking their face or hand	N/S	Catala et al. (114)

		-Yorkshire Terrier<br><br>-Beauceron<br><br>-Border Collie<br><br>-Australian Shepherd<br><br>-German Shepherd<br><br>-Bernese Mountain Dog					
SADs	Experimental	19 pet dogs (68.40% pedigree and 31.60% mixed breeds)	From 6 to 185 months	-untrained	- Maintained eye contact with the owner<br><br>-Pawing<br><br>-Nudging<br><br>- Stay near the owner	N/S	Powel et al. (112)
SRDs and SADs	Anecdotal	22 pet dogs:<br><br>-Golden Retriever<br><br>-Standard Poodle<br><br>-German Shepherd<br><br>-Akita<br><br>-Rough Collie	N/S	-Trained and untrained	- Licking<br><br>- Decreased motor activity<br><br>- “protective” behavior without aggression<br><br>- Whimpering	Median accuracy of 80%	Kirton et al. (18)

		-Rottweiler<br><br>-Terrier<br><br>-Great Pyrenees<br><br>-Mixed breeds					
SADs	Case reports	2 pet dogs and one horse	N/S	Trained	-Sitting<br><br>-Barking<br><br>- Kicked in the head by the horse<br><br>- Standing up<br><br>-Staring	N/S	Ortiz and Liporace (115)
SADs	Case reports	-Labrador retrievers<br><br>-Jack Russell<br><br>-Border collie<br><br>-Miniature schnauzer<br><br>-Collie	From 8-month-old to 5-year-old	Trained (6 months)	-Bark<br><br>-Jump up<br><br>-Pawing	N/S	Strong et al. (82)

Supplementary table 1. Abbreviatures: (N/S) Not Specified; (DADs) Diabetic-Alert-Dogs; (MDD) Medical Detection Dogs; (SAD) Seizure-Alert Dogs; (SRD) Seizure-Response Dogs.

Supplementary table 2.

Patient data (disease, age, sex)	Animal data (species, breed, age)	Activities	Time o period	Parameters	Outcomes	Reference
Pediatric heart transplant patients (aged 6–19 years).	NA	Walking, petting, and grooming	One week with therapy sessions averaging 17.2 min	-Blood pressure<br>-Respiratory rate<br>-Ambulation data	↓ Systolic and diastolic blood pressure<br>↑ Walked	Walden et al. (127)
Children with a diagnosis of a solid tumor or acute lymphoid leukemia (6 – 12 years)	Retriever and a golden retriever dog	- Brush, pet, and play fetch with the dog<br>- Give water and food to the food<br>-Walking with the dog<br>-Dog show; playing with the dog's supplies; dog drawing	Three sessions of 30 min	-Blood pressure<br>-ESI<br>-AUQEI<br>-Child Depression Inventory<br>-BRUMS<br>-Faces Pain Scale<br>-AAT Assessment Questionnaire	↓ Blood pressure<br>↓ ESI<br>↓ BRUMS<br>↓Faces Pain Scale	Silva and Osório (20)

Adult oncological patients (~64.5 years)	Two trained dogs:<br><br>8-year-old male border collie<br><br>9-yearold female Shetland sheepdog	-Observed a dog doing some exercises<br><br>-Play with a dog<br><br>-Hold a dog and/or feed it	-During weekly chemotherapy sessions (out once a week)	-Anxiety<br><br>-Depression<br><br>-Somatic Symptoms<br><br>-Hostility test<br><br>-HR<br><br>-Blood pressure<br><br>-Arterial oxygen	↓Anxiety levels<br><br>↓Aggressiveness<br><br>↓HR<br><br>↑Arterial oxygen	Orlandi et al. (4)
Male patient hospitalized with colon cancer, lumbar abscess, and diabetes mellitus (79-year-old)	N/S	- Interact with the patient	-Four sessions (15 minute-visit by the dog/handler in each session)	-Facial expressions<br><br>-Verbal communication<br><br>-Eye contact	↑ Verbal communication	Aiba et al. (144)
24 patients with chronic schizophrenia (~age of 47.8 years)	5 trained dogs	- Handling the dog<br><br>-Walking<br><br>-Training<br><br>-Playing with the animals	- Six months (twice weekly for 1 hour)	-PANSS<br><br>-QoL<br><br>-EQ-5D<br><br>-Cortisol<br><br>- sAA	↓PANSS<br><br>↓Cortisol<br><br>↑sAA	Calvo et al. (146)
40 middle-aged and older adults	4 dogs (5-11 years old):	-Physical (handling,	12-week (1 h weekly)	-MoCA<br><br>-CST	↓CST<br><br>↓ACIS	Chen et al. (151)

	-Labrador<br><br>-Corgi<br><br>-Maltese<br><br>-Shiba Inu	feeding, grooming)<br><br>-Cognitive (training and orienting the dog)<br><br>-Social (talking to the dog)<br><br>-Sensory (touching, playing) activities		-TUG<br><br>-5MWT<br><br>-ACIS		
10 elderly schizophrenic patients (mean age 79.1 years)	N/S	-Petting<br><br>-Feeding<br><br>-Grooming<br><br>-Bathing them	12 months (Weekly 4-hour sessions)	-SAFE<br><br>-ADLs	↓ SAFE<br><br>↑ ADLs	Barak et al. (152)
Older adults (~65 years old) with and without depression	-Dogs<br><br>-Cats<br><br>-Birds	-Stroking the animal<br><br>-Playing with the animal<br><br>-Throwing and retrieving balls	10-90 minutes three times per week (6 weeks to 8 months)	-GDS<br><br>-CSDD	↓GDS	Villareal-Zegarra et al. (23)

		-Playing with the animal and even coexistence between the two				
58 patients diagnosed with depression (~60–70 years old)	-Trained dogs	-Dog handler	2–3 h per day over a 3-week period	-BDI-9<br>-STAI<br>-WBI<br>-IIRS<br>-VAS<br>-QoL	↓BDI-9<br>↑STAI<br>↑WBI<br>↓IIRS<br>↓VAS<br>↑QoL	Mittly et al. (158)
15 adolescents with attention-deficit hyperactivity disorder and severe depressive symptoms	-Horses	-Caring it<br>-Feeding it<br>-Brushing it<br>-Therapeutic riding	Two 60-minute sessions over seven days	-Functional brain connectivity<br>-CDI	↑Brain functional connectivity (left and right amygdala)<br>↓CDI	Kang et al. (160)
80 Children (aged 5-17 years) with suspected moderate to high anxiety	Dogs trained	-Handler	10 minutes	-FACES<br>-Salivary cortisol	↓FACES	Kelker et al. (25)

83 patients (<40 to >60 years old)	Dogs	-Freely able to touch	15 minutes	-FACES Anxiety<br><br>-Pain<br><br>-Depression	↓FACES Anxiety<br><br>↓Pain<br><br>↓Depression	Kline et al. (26)
60 children (6–17 years) with depression	Horses	-Riding	10 sessions of one-hour adaptive riding sessions	-SCARED<br><br>-ERC<br><br>-SEQ-C	↓SCARED<br><br>↓ERC<br><br>↓SEQ-C	Hoagwood et al. (166)
249 university students (~19.94 years old) with depression	-16 adults pet dogs<br><br>-14 cats	- Students freely interacted with animals, engaging in petting and stroking.	10-minute animal visitation	-Salivary cortisol	↓ Cortisol levels	Pendry et al. (7)
21 dementia patients with a mean age of 84.7 years	-Three Golden Retrievers and a Pincher	-Hold, stroke, walk, talk, and play with four dogs	90 minutes once a week for six weeks	-MMSE<br><br>-GDS	↑MMSE<br><br>↓GDS	Moretti et al. (178)
50 Alzheimer's patients (~75 years old)	A 7-year-old female Labrador retriever	Interaction with the dog (attention: fetch, hide the ball, caring for the dog)	Once a week for 45 min over 6 months	-MMSE<br><br>-GDS	↓MMSE<br><br>↓GDS	Menna et al. (6)

Institutionalized patients 65 years of age and older	8 trained dogs:<br><br>-Golden retrievers<br><br>-Flat-coated retriever	-Verbal interaction with the dog<br><br>-petting or stroking the dog<br><br>-Throwing the dog a bit of food or a toy<br><br>-Tapping, touching, or carrying out an act directed at the dog handler	10 weeks, patients participated in individual 30-min sessions	-GDS<br><br>-MMSE<br><br>-GAD-7<br><br>-PANAS<br><br>-IPQ-R<br><br>-NPRS	↓GDS<br><br>↓NPRS	Ambrosi et al. (24)
100 residents in nursing homes (~85.5 years old)	-Two untrained dogs (3 to 8 years old):<br><br>Retrievers or retriever mixes<br><br>-Robot seal (PARO)<br><br>-Soft toy cat	-Interact with the 'animal' both verbally and by touching it	Biweekly visits for 6 weeks	-MMSE<br><br>-GDS<br><br>-CAM<br><br>-Sleep data<br><br>-Weight and height	↓MMSE<br><br>↑GDS<br><br>↑Sleep time	Thodberg et al. (183)
12 autistic children (5 to 10 years old)	-Dogs	-touching, holding, and petting activities	18 sessions (20 minutes long)	-Autistic behaviors (handing postures, humming, spinning	↓ Autistic behaviors	Redefer and Goodman (192)

		-feeding, ball-throwing, bubble blowing, and grooming		objects, and repetitive jumping)<br><br>-Social interaction	↑ Social interaction	
Children (12–18 years old) diagnosed with ASD	Labrador and Labradoodle breed	-Small exercises and performing certain commands of the dog	Six weekly sessions	Behavioral synchrony and CBCL	↑Proportion of synchrony<br><br>↓CBCL	Griffioen et al. (196)
Children from 4 to 19 years, diagnosed with ASD	15 trained dogs	-Brushing, feeding, walking, and playing with the dog	One hour once a week for five weeks	-Child’s interactions and communications<br><br>-Confidence<br><br>-Motivation<br><br>-Emotional regulation	↑ Child’s interactions and communications<br><br>↑ Confidence<br><br>↑ Motivation<br><br>↑ Emotional regulation	London et al. (197)
Children (7–17 years) diagnosed with ASD	Labrador and Poodle dog breeds	Complete an obstacle course together	From 4 to 6 weeks	-Social confidence<br><br>-Social motivation<br><br>-Emotional attunement<br><br>-Emotional regulation	↑ Emotional attunement<br><br>↑ Emotional regulation	Van Der Steen et al. (198)

20 children with ASD (6–14-year-olds)	Two therapy dogs (Boxer breed)	-Warm-up<br><br>-Circuit of agility activities<br><br>-Cool-down	Weekly physical activity session for seven weeks (60 min)	-Time of light, moderate to vigorous physical activity<br><br>-Sedentary time<br><br>-Estimated bone impacts	↑Light physical activity<br><br>↓ Sedentary time	Abadi et al. (194)
10 Children with ASD (2.5 to 6.5 years old)	Clumber Spaniel, Newfoundland, and Border Collie/Yellow Lab cross	-Providing a ball and a stuffed dog to children	Three times per week for a total of 15 weeks	-Behavioral<br><br>-Verbal responses	↑Laughing<br><br>↑ Talked about themselves<br><br>↑ Talked more to the dog	Martin and Farnum (27)
Boy diagnosed with ASD (12-year-old)	Female Labrador retriever (trained)	- one-to-one activities	- Each week (for 45 minutes)	-Positive behavior<br><br>-Positive physical contact<br><br>-Aggressive behaviors	↑Smiling<br><br>↑ Positive physical contact<br><br>↓ Aggressive behaviors	Silva et al. (200)
Children with ASD (ages 8–14)	S/N	S/N	- 12 weeks of weekly treatment	- Functional Problems subscale<br><br>-Social deficits<br><br>-Social communication	↓ Functional Problems subscale<br><br>↓ Social deficits<br><br>↑ Social communication	Becker et al. (28)

				-Repetitive behaviors	↓ Repetitive behaviors	
Three preschool children (two boys and a girl, aged 7-8 years) with ASD	A Labrador dog (2 years old)	-Freely interact with the dog (i.e., greet, touch, or hug the dog	-Three h/day therapy (two sessions per week)	-Appropriate Social Interactions<br><br>-Relevant to the Target Social Skill<br><br>-Level of Prompt Needed to Provide the Expected Social Response<br><br>- Frequency of the Initiations of Social Interactions	↑ Appropriate Social Interactions<br><br>↑ Relevant to the Target Social Skill<br><br>↓ Level of Prompt Needed to Provide the Expected Social Response<br><br>↑ Frequency of the Initiations of Social Interactions	Grigore et al. (202)
10 autistic children (aged 7–10)	Two Golden Retrievers	- Take care of them, for example, by combing their hair and feeding them.<br><br>-Walk the dogs and throw balls to them	-14 individual sessions	-Verbal social behavior	↑ Verbal social behavior	Fung et al. (191)

Adults with ASD (18–60 years)	Labradors, Labrador crossbreeds, Golden retrievers, Poodles, and German Wirehaired Pointer dogs were used	-Free interaction with dogs	Individual sessions for a 10-weeks	-Perceived Stress Scale<br><br>-Social Responsiveness Scale	↓ Perceived Stress Scale<br><br>↓ Social Responsiveness Scale	Wijker et al. (29)
6 high-functioning adults with ASD (From 23 to 54 years old)	Two poodles and two Labrador crossbreeds (trained)	-Observation and interpretation of the behaviors of the dog<br><br>-Basic instructions<br><br>-Guiding the dog without a leash through an obstacle course<br><br>-Outdoor walking with the dog	10 weekly one-on-one sessions	-Rosenberg Self-Esteem Scale	↑ Rosenberg Self-Esteem Scale	Wijker et al. (207)
25 adults (36 to 57 years) with ASD	S/N	S/N	Twice a week for 10 weeks	-Tinetti test<br><br>-Balance	↑ Tinetti test<br><br>↑ Balance	Fernández-Sánchez et al. (210)

				-Risk of falling	↑ Risk of falling	
24 adults older than 40 years old, diagnosed with ASD	2 Labrador retrievers (5 and 11 years old)	Climbing stairs, walking, and moving through an obstacle course	Two sessions per week for 10 weeks	-Walking performance<br><br>- Balance<br><br>-Risk of falling<br><br>-Communication abilities<br><br>-Ability to climb and descend stairs	↑-Balance and Risk of falling<br><br>↑ Walking performance	Gómez-Calcerrada et al. (212)
39 Autistic adult (37.1612.1 years old)	-Two Bernese Mountain Dogs<br><br>-One Bergamasco Shepherd<br><br>-One French Bulldog<br><br>-Two medium-sized, mixed-breed dogs	-Taking care of the dog<br><br>-Grooming<br><br>-Interactive playing<br><br>-Driving the dog on a leash	20-session one a week	-Cognitive domains	↑ Attention to movement<br><br>↑ Visuomotor coordination<br><br>↑ Exploration play<br><br>↑ Motor imitation<br><br>↑ Social skills	Scorzato et al. (213)
28 Children with ASD (aged 6–12 years)	Twenty specially-trained horses (different	-Grooming, hand walking,	Six-month period	-Problem-solving tasks	↓ Problem-solving tasks	Borgi et al. (214)

	breeds, medium size)	and horseback riding		-Vineland Adaptive Behavior Scale	↑ Vineland Adaptive Behavior Scale	
children with ASD (aged 9–14 years)	Horses	- Riding activities	S/N	- Social Responsiveness Scale<br><br>-Sensory profile	↑Communication<br><br>↑ Affective state<br><br>↑ Social attention<br><br>↑ Physical movements<br><br>↑ Dynamic motor skills	Măierean et al. (215)
34 children (aged 5–10 years) with ASD	Horses	-Riding backhorses<br><br>-Walk, trot, and halt on their horse	-12-week horseback riding intervention	-Social Responsiveness Scale<br><br>-Sensory profile	↑ Social Responsiveness Scale<br><br>↑ Sensory profile	Bass et al. (216)
127 participants with ASD (ages 6–16 years)	Horses	- helmet and mounted a horse	- 10-week interventions were a minimum of 45 minutes	-ABC-C scale<br><br>-Self-regulation<br><br>-Socialization<br><br>-Communication	↓ Irritability<br><br>↓ Hyperactivity scores<br><br>↑ Social cognition	Gabriels et al. (30)
240 autistic children (6–12 years)	Horses	-Riding backhorses	45–70 min sessions of horse-assisted riding,	-Social function	↑ Social skills	Chen et al. (184)

			1–2 times per week, for 7–24 weeks	-Aberrant behavior		

Supplementary table 2. Abbreviatures: (N/S) Not Specified; (ESI) Child Stress Symptoms Inventory; (AUQEI) Quality of Life Evaluation Scale; (BRUMS) Adapted Brunel Mood Scale; (HR) Heart rate; (PANSS) Positive and Negative Syndrome Scale; (QoL) quality of life; (EQ-5D) Health-Related Quality of Life Questionnaire; (sAA) Alpha-amylase; (MoCA) Montreal Cognitive Assessment; (CST) Chair Stand Test; (TUG) Timed Up-and-Go; (5MWT) 5-Meter Walk Test; (ACIS) Assessment of Communication and Interaction Skills; (SAFE) Social Adaptive Functioning Evaluation Scale; (ADLs) activities of daily living; (GDS) Geriatric Depression Scale; (CSDD) Cornell Scale for Depression in Dementia; (BDI-9) Beck Depression Inventory; (STAI) Spielberger State Anxiety Inventory; (WBI) WHO Well-Being Index; (IIRS) Illness Intrusiveness Rating Scale; (VAS) Visual Analogue Scale; (CDI) Child Depression Inventory; (SCARED) Screen for Child Anxiety Related Disorders; (ERC) Brief Experiences in Close Relationships; (SEQ-C) Self-Efficacy Questionnaire for Children, Emotional Self-Efficacy Subscale; (MMSE) Mini-Mental State Examination; (GAD-7) Generalized Anxiety Disorder 7; (PANAS) Positive and Negative Affect Schedule; (IPQ-R) Illness Perception Questionnaire-Revised; (NPRS) Numeric Pain Rating Scale; (CAM) Confusion Assessment Method; (ASD) autism spectrum disorder; (CBCL) Child Behavior Checklist; (ABC-C scale) the Aberrant Behavior Checklist;
